# Adsorbent Materials Based on Modified Chitosan for Purification of Aqueous Media from Pharmaceutical Residues, Primarily Antibiotics

**DOI:** 10.3390/polym17192601

**Published:** 2025-09-26

**Authors:** Balzhima Shagdarova, Yulia Zhuikova, Alla Il’ina

**Affiliations:** Institute of Bioengineering, Research Center of Biotechnology of the Russian Academy of Sciences, Moscow 119071, Russia; zhuikova.uv@gmail.com

**Keywords:** adsorption, chitosan adsorbent, water purification, micropollutants, pharmaceuticals, antibiotics

## Abstract

This literature review highlights the latest advances in the use of adsorption materials based on modified chitosan for the purification of aqueous solutions from pharmaceutical residues. Some countries are actively working to detect pharmaceuticals and their metabolites in water samples from natural sources and municipal wastewater, as well as to study their impact on the environment. In this article, adsorbents based on chitosan, a natural, low toxic and biodegradable polymer, are considered as a promising solution to this problem. Due to some disadvantages of pure chitosan (low mechanical strength, small specific surface area), its practical application is limited. One of the ways to overcome them is to create modified materials, such as grafted copolymers, as well as chitosan derivatives and its composites, including those with magnetic nanoparticles and carbon materials. Modification of chitosan makes it possible to achieve an increase in mechanical strength, specific surface area and porosity. The high efficiency of hybrid adsorbents is emphasised, demonstrating high adsorption capacity, reuse ability and selectivity for a wide range of pharmaceutical preparations, including antibiotics. Thus, despite a number of limitations, chitosan-based materials are a promising solution for deep wastewater treatment.

## 1. Introduction

The main indicator that characterises the environmental safety of the habitat of all mankind on the Earth is the quality of natural waters [1]. Pharmaceuticals, their metabolites, personal care products, and agrochemicals are a large group of chemicals that are classified as micropollutants [2,3,4]. In addition, the main pharmaceutical environmental pollutants can be identified: medicines that do not comply with regulations (pharmaceutical waste), expired drugs, medicines that have lost their essential consumer properties, and preparations for animals.

**Figure 1:**
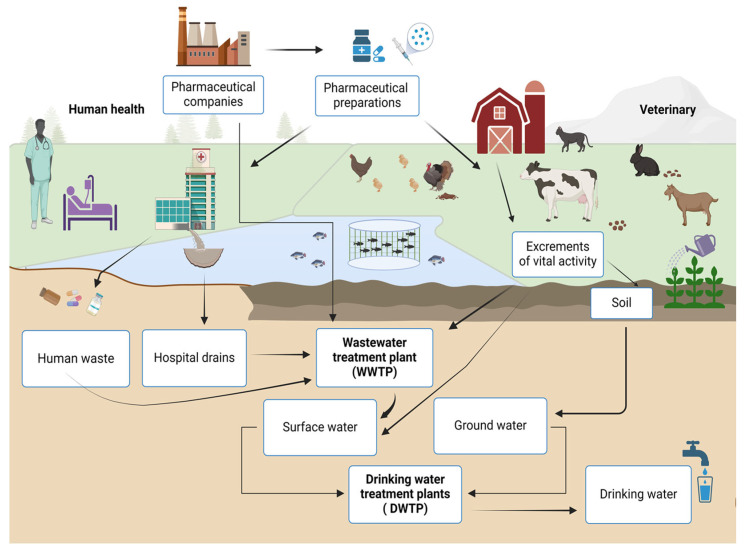
Scheme of pharmaceutical products and their metabolites in the environment and water purification.

Today, there is a wide range of therapeutic drugs that are used to treat or prevent human and animal diseases. Their presence in the environment is not sufficiently assessed, which is a cause for concern [5]. The reasons for the negative consequences are the expansion of the production of pharmaceuticals, the increase in the range of pharmaceuticals, and their active use all over the world for the treatment of long-known and new diseases. Currently, the disposal of expired pharmaceuticals is insufficiently established; often, they are simply thrown into the environment, including after the expiry date. In addition, they are excreted from the body with human physiological secretions as a result of incomplete metabolism. The result is a mixture of compounds in the wastewater. The main sources of pollution are (Figure 1) as follows:

- Wastewater from enterprises that use insufficient purification methods.
- Wastewater from agricultural enterprises that use medicines.
- Domestic sewers with biological fluids of people who take medications.
- Leaching of waste disposal sites.
- Disposal of pharmaceutical waste by imperfect methods.

The full impact remains unclear on how the presence of pharmaceuticals and their metabolites in water can affect human health and the environment. A significant proportion of pharmaceutical waste enters the aquatic environment. Their detectable low concentrations, ranging from ng/L to g/L, can persist for long periods of time and become biologically active as they accumulate [6]. The researchers conducted a thorough analysis [7] on the presence of pharmaceutical preparations in various types of water, below are data on the example of different classes of drugs—sulfonamides, non-steroidal anti-inflammatory drugs, and anticonvulsant (Table 1).

Inevitably, a number of issues arise, among which the quantification of pharmaceutical residues and metabolites in wastewater treatment plant effluents is of great importance. This is necessary to assess the degree of hazard to the environment, including humans. 

## 2. Presence of Pharmaceutical Preparations in the Wastewater of Several Countries

Studies conducted in many countries indicate their prevalence, with the presence of micropollutants detected in aquatic ecosystems [8,9,10].

Environmental risks from the use of pharmaceuticals are known in some countries. Twenty-three pharmaceuticals in water samples at different stages of conventional and natural wastewater treatment plants (WWTPs) were quantified, and the environmental risk from their occurrence in wastewater in Gran Canaria (Spain) was assessed [11]. All studied compounds, except omeprazole, were detected in most sampling points, in a concentration range from 0.004 ± 0.001 to 59.2 ± 11.7 μg/L from conventional WWTP1 and from 0.018 ± 0.001 to 148 ± 14.7 μg/L from natural WWTP2. A simplified solid-phase extraction methodology was used for the quantification of the target pharmaceuticals. It is based on a three-step protocol (excluding conditioning and equilibration steps) and involves a combination of liquid chromatography and mass spectrometry (LC-MS/MS) techniques [12]. Conventional purification gave a high yield in removing the target pharmaceuticals, and the efficiency was more than 80%. Conventional purification showed high purification efficiency with a median removal of 99.7% of target pharmaceuticals. The exceptions were ibuprofen (IBU), carbamazepine (CBZ), and fluoxetine (FLX) with removal efficiencies of 40%. These compounds pose an ecological risk to many aquatic organisms (algae, daphnia, and fish). The authors believe that such assessments could be used to guide future research to prioritise the use of particular pharmaceuticals.

In the work [13], the overall ecological risk potential of the used pharmaceuticals. These pharmaceuticals were present in significant concentrations in the wastewater of a general hospital and a specialised treatment centre in Switzerland was investigated and assessed. Information on the concentrations of the drugs, which were found in the studied wastewater, was compared with the literature data on human excrement. Experimental data were available for less than 20% of the 100 pharmaceuticals at the highest patient intake load. The effect assessment was performed using quantitative structure–activity relationships. The authors took into account that many pharmaceuticals are acids or bases, which completely or partially dissociate at the pH of the environment. For this purpose, a correction for changes in their structure was introduced into the toxicity prediction model. The risk quotient (RQ) for release to wastewater was found to be dominated by diclofenac, ritonavir, clotrimazole, and amiodarone. It was defined as the ratio between the predicted environmental concentration and the concentration at which no effect is expected. A numerical value of RQ > 1 indicates an ecotoxicological risk to the aquatic environment, and RQ value < 1 indicates no risk. Data are limited for the three compounds with the highest risk, ritonavir, clotrimazole, and amiodarone, which were used in a general hospital. Ecotoxicological effects have only been well studied for diclofenac. In one specialised treatment centre, diclofenac was among the three riskiest, along with ritonavir and clotrimazole. It can be assumed that additional wastewater treatment at these health centres should reduce the pharmaceutical load of hazardous pharmaceuticals to the environment.

The human health hazard and environmental risk assessment of nine pharmaceuticals present in water samples from three rivers in Malaysia were investigated and evaluated [14]. Examination of the physical and chemical properties of the water samples showed that they belong to the second class of national water quality standards. This means that additional treatment is required before consuming this water. The investigated pharmaceuticals—ciprofloxacin (CIP), nitrofurazon, sulphamethoxazole (SMX), caffeine, chloramphenicol, diclofenac, triclosan, amoxicillin, and dexamethasone—were detected in the river water of three rivers at the sampling locations. The exceptions were dexamethasone, diclofenac, and triclosan, which were not detected at some sampling sites. Analysis and quantification were performed using commercially available enzyme immunoassay kits. The concentration of CIP was the highest at 299.88 ng/L. This is probably due to the fact that CIP was listed as the most commonly used antibacterial agent in Malaysia in 2009 and 2010 (Ministry of Health data, 2014). The average concentration of diclofenac in the three rivers was 2.76 ng/L, 4.84 ng/L, and 4.30 ng/L. Diclofenac is the most commonly used drug in Malaysia in 2010 (Ministry of Health data, 2014). Focusing on human health hazard quotient—its values were less than one, and for CIP and dexamethasone, between 1.521 and 41.36. The ecotoxicological hazards, which were determined using RQ values, were within moderate risks, except for the values for diclofenac (RQ greater than 30). For the drugs listed above, the results obtained are comparable with the literature data. To adjust the results, the authors suggest using gas chromatography mass spectrometry and physical separation capabilities using (LC-MS/MS) [15]. Based on the results obtained, researchers believe that pharmaceutical production, purification technology, and treated wastewater are sources of pharmaceuticals in surface water samples.

A systematic method for screening of suspected pharmaceuticals and their metabolites was developed. This method quantifies the residues in water samples from lakes, rivers, and urban wastewater of Wuhan City, China [16]. Residues were quantified by solid-phase extraction followed by liquid chromatography and high-resolution mass spectrometry. A database of approximately 250 drug names without reference standards was created for drug identification. More than 30 drugs were identified without the use of reference standards. Out of 29 identified drugs (8 antibiotics, 9 metabolites, and 12 miscellaneous drugs), 26 compounds (90%) were identified by the screening method. This showed that the screening method for putative compounds was highly effective. According to the authors, out of 33 identified pharmaceuticals, 6 compounds are caffeine, metformin, theobromine, valsartan, metoprolol acid, and dipyrone. Dipyrone is rapidly hydrolysed to the 4-AA (C_11_H_13_N_3_O), 4-AAA (C_13_H_15_N_3_O_2_), 4-FAA (C_12_H_13_N_3_O_2_) metabolites, the concentration of which is more than 100 ng/L. In addition, a high frequency of occurrence was observed in the surface water of Wuhan. The removal rates of caffeine, metformin, theobromine, and valsartan in municipal wastewater treatment plants were high, while those for the metabolites dipyrone-4-AAA and metoprolol acid were low. The authors noted that the sources of drinking water consumed were the Yangtze and Han rivers, which were disinfected before use by residents. A cause for concern is that disinfection by-products are more toxic than pharmaceuticals. Many pharmaceuticals cannot be completely removed in wastewater treatment plants; they are usually detected at low concentrations of ng/L to µg/L. Advances in analytical methods and more sensitive equipment make it possible to detect low levels of pharmaceuticals and their metabolites in the aquatic environment. Long-term monitoring of pharmaceuticals in the aquatic environment usually indicates their accumulation.

Paíga et al. [8] proposed the use of solid-phase extraction followed by ultra-high-performance liquid chromatography with tandem mass spectrometry (SPEUHPLC-MS/MS) to analyse surface water and wastewater from treatment plants. The therapeutic classes investigated included non-steroidal anti-inflammatory drugs/analgesics, antibiotics, and psychiatric drugs. The main results obtained from the wastewater samples indicate the presence of a large number of pharmaceuticals. In 2013/2014, the company sampled water from the Lis River and two wastewater treatment plants (effluent and influent) to assess the presence of pharmaceuticals. The 2018/2019 studies aimed to analyse the same samples. The cumulative concentration of samples analysed from 2018 to 2019 was higher than in 2013 and 2014. The highest concentrations (µg/L) in both years 2018–2019 were identified by sampling companies for hydroxybuprofen, diclofenac, ibuprofen, naproxen, atenolol, and caffeine. For example, caffeine concentrations ranged from the method’s lower detection limit (<MDL) to 3.20 µg/L in 2018 and from <MDL to 53.0 µg/L in 2019. The authors believe that wastewater treatment plant effluent monitoring is necessary for the identification and release of priority pharmaceuticals. This is important for assessing the need for additional (quaternary) treatment as required by the new European Union Urban Wastewater Treatment Directive [17]. For example, for most of the analysed samples, the sum of concentrations between 2017 and 2019 was higher than the results obtained in 2013 and 2014. There was a tendency for their potential accumulation over time. The authors noted that most published studies focus on the parent compounds, and less attention is paid to determining the concentrations of metabolites or degradation products of the drugs.

A method was developed for the detection of some sulfonamide antibiotics—SMX, sulfadiazine (SFD), sulfapyridine (SFP), sulfamethazine, sulfathiazole, sulfamerazine, sulfamethizole, and sulfisoxazole in water samples (target analysis) [18]. Water samples were collected from artificial lakes, local rivers, and streams at four sites in the most urbanised area of Poland. The analyses were performed using UHPLC-MS/MS. The most frequently detected contaminants in surface water were SMX and SFP with maximum concentrations of 78.88 ng/L and 38.88 ng/L, respectively. Trace amounts of SFD, 0.40 ng/L, were detected. Due to the long residence time in such water bodies, there is a high probability of accumulation of pollutants. A non-targeted analysis was performed by solid-phase extraction. Next, ultra-high-performance liquid chromatography was used in combination with tandem mass spectrometry (SPE-UHPLC-MS/MS). The purpose of the study was to identify transformation products in order to find out the ways of their degradation. Several SFD and SMX transformation products were detected and confirmed in environmental samples. Antibiotic transformation products constitute a distinct, broad group of compounds that are potentially hazardous due to their unknown structures and physicochemical properties. It is known that they can exhibit high biological activity and toxicity compared to the corresponding parent compounds [19].

The results of the studies thus confirmed the presence and prevalence of water contamination with antibiotics in most cases and the correlation of the problem with ecology. Reliable quantification of pharmaceutical residues and metabolites in different environments is necessary to have an idea of the degree of risk to humans and biota. Pharmaceuticals can persist in the surrounding surface water even at low concentrations. Little is known about their long-term effects on human health and aquatic organisms; some cause endocrine disruption. A review [5] summarised the results of nearly 1000 articles, reports, and other publicly available documents published between 2014 and 2016 on the presence of pharmaceutical residues and metabolites in environmental objects. The need for additional treatment to effectively remove these compounds is clear.

## 3. Methods Used to Remove Pharmaceutical Contaminants in Water Treatment Systems

Methods of removing pharmaceutical contaminants in water treatment systems are different. In most cases, the technologies used include: membrane filtration, electrolysis, forward and reverse osmosis, ion exchange, adsorption on activated carbons, or advanced oxidation processes using ozone, ultraviolet radiation, or gamma radiation [20,21].

When using forward osmosis technology for wastewater treatment, there are several problems, such as concentration polarisation, membrane fouling, and reverse diffusion of dissolved substances. For commercial application of the technology, it is necessary to develop new membrane materials with improved characteristics. This will increase the extraction efficiency and reduce the energy consumption of the process [22]. Reverse osmosis technology also plays an important role in the treatment of industrial wastewater. However, the disposal of concentrate using the reverse osmosis process is a difficult task due to the high content of organic pollutants.

In the process of biological treatment using sludge, the removal mechanism depends on the nature of the pharmaceutical and its properties (e.g., hydrophobicity and biodegradability), the type of sludge, etc. The removal of pharmaceuticals varies depending on the contribution of biological degradation and sorption as key mechanisms for the removal of pharmaceuticals [23,24].

Advanced oxidation processes are alternative treatment methods that allow various types of radicals (e.g., HO•) to be generated and used to break down pollutants [25]. These processes include microelectrolysis and Fenton oxidation [26]. Electrolytic cells have advantages such as ease of process automation, modular reactor design, and the ability to adapt to changing organic matter content in wastewater, including the absence of the need for storage and processing of chemicals. However, the use of electrochemical treatment on an industrial scale is extremely small. This method can lead to an increase in the toxicity of water during the formation of chlorinated by-products [27]. The photo-Fenton process is a powerful and effective Advanced Oxidation Process (AOP) that uses light to enhance the classic Fenton reaction, allowing for the faster and more complete destruction of hazardous pollutants in water. The use of photo-Fenton and ozonation processes is a step towards the effective removal of pharmaceuticals. Intensive ozonation using UV radiation, which has a high ability to neutralise pollutants, activates the formation of radicals [28].

Membrane technologies and adsorption are the methods most commonly used to remove pharmaceuticals, including antibiotics. These will be discussed in more detail below.

### 3.1. Membrane Technologies

Membrane technologies use semi-permeable membranes that selectively allow certain components to pass from the treated water media, including for the removal of pharmaceuticals. The separation is based on differences in the rates of diffusion of molecules through the membrane material. The advantages of membrane technologies include: compactness and accessibility, low power consumption with small volumes, continuity of operation, ease of scaling, and technology security [29].

Membrane technologies appear promising for removing pharmaceuticals from the aquatic environment [30]. However, in a critical review [31], the authors consider that despite the high removal efficiency of the compounds, the main disadvantage of membrane separation is the increase in their concentration, i.e., further treatment of the concentrate by micro- and ultrafiltration processes is required.

The combined conventional and advanced ultrafiltration and reverse osmosis treatment of drinking water has been shown to remove 28 pharmaceuticals and 12 narcotic substances with an efficiency of 94%. The compounds that were detected in the treated water were iopromide (up to 17.2 ng/L), nicotine (13.7 ng/L), benzoylecgonine (1.9 ng/L), cotinine (3.6 ng/L), acetaminophen (15.6 ng/L), erythromycin (2.0 ng/L), and caffeine (6.0 n/L). The compounds were analysed by liquid chromatography coupled with UPLS-MS/MS mass spectrometry using an Acquity BEH C18 column. The researchers believe that the presence of the compounds, in thoroughly purified water, even at such low concentrations, as well as their transformation products, implies more information and further study [32].

In a review [33], it highlighted the removal of micropollutants from aqueous solutions by membrane separation. It was found that the process of membrane separation of micropollutants was carried out in most of the works with a treatment index of more than 90%. It was observed that the best removal efficiency of micropollutants is possible at pH values above the pKa of the compound, which is due to the electrical repulsion forces when they hit the membrane. The removal efficiency of micropollutants by membrane separation can be related to the size of the molecules. Advanced oxidation processes—electrochemical oxidation, ultrasonic irradiation, and ozonation, etc.—are used for further purification of the concentrated waste stream after the membrane separation stage. These processes exploit the high reactivity of hydroxyl radicals for the gradual oxidation of organic compounds to presumably harmless products. In addition, it has been observed that the degradation of micropollutants during oxidation has contributed to the formation of by-products that may be active and toxic to living organisms and the environment. In order to develop effective methods for the removal of micropollutants, it is necessary to know their physicochemical properties and to use the available treatment methods and conditions. It should be noted that reverse osmosis and membrane filtration are not widely used for the subsequent treatment of drinking water due to the economic disadvantage of these methods.

Wastewater treatment plants, which consist of a primary physicochemical treatment system and a secondary system, most often a biological activated sludge reactor, have a limited capacity to remove pharmaceuticals from municipal wastewater. This is because their biodegradation by microorganisms occurs by co-metabolism (in which case other substances are carbon or energy sources during transformation) or by substrate consumption (in which case the drug is a carbon and energy source). In addition, medicinal products may inhibit microbial activity or mineralisation (i.e., conversion of the contaminant to carbon dioxide, water, and inorganic ions), or degradation to smaller/shorter-chain products may occur. The formation of metabolites or biotransformation during the biological processing of pharmaceuticals depends on specific conditions, such as the nature of the drug and the type of microorganisms [20,34,35,36].

The process of purification from pharmaceuticals and their metabolites using a membrane biological reactor was analysed [23]. The improved membrane bioreactor technology may include the use of activated sludge and the selective separation characteristic of membrane processes. Comparative results of the average removal efficiencies of pharmaceuticals in the activated sludge and membrane bioreactor processes and the mechanism of their removal by biodegradation and sorption are presented. For example, the removal efficiencies of diclofenac and ketoprofen using the membrane bioreactor were 32 and 99 per cent, while those using activated sludge were 99 and 50 per cent, respectively. The removal process of diclofenac implied biodegradation in the range of 5–45% and sorption less than 5%, while for ketoprofen it was 70%, mainly due to biodegradation, while sorption was completely absent. Both drugs are active environmental pollutants due to their stability in the aqueous environment. Therefore, the use of a membrane bioreactor is one of the most efficient purification methods. However, membrane fouling and rewashing are factors that limit the use of the process on a large scale. Research into the development of optimised membrane bioreactor technology is therefore contributing to the more complete removal of micropollutants.

It is believed that membrane properties should be improved to reduce membrane fouling. Designing membranes from nanomaterials could be one way to improve their properties and performance. In a review [37], the authors discussed studies on the use of nanomaterial membranes based on their structural morphology. For each type, their design and fabrication, as well as their potential applications, were discussed. To date, various nanomaterial-based membranes, including nanofibres, nanoparticles, nanotubes, nanocrystals, nanowires, and nanosheets, have been used for water purification. Nanomaterial-based membranes are more effective than conventional materials. This concerns their hydrophilicity, surface roughness, thermal stability, hydraulic stability, fouling control, higher water permeability, and selectivity due to small surface pore sizes. The use of technology incorporating nanomaterials in a membrane biological reactor shows good performance. However, the authors believe that there are aspects that are still poorly understood. These include the cost of large-scale production of such membranes, their lifetime in full-scale use, the leachability of nanomaterials, and their presence in wastewater, sludge, etc. These issues should be further investigated before nanomaterial-based membranes are introduced into membrane bioreactor technology.

Membrane technologies have some disadvantages. One of them is the limited selectivity; without additional purification, it is difficult to achieve a purity of more than 90%. In addition, the membrane may be damaged by mechanical impurities and reagents. The consequence of this is a decrease in membrane performance over time. There are also restrictions on the operation of membranes in terms of temperature and pressure.

It is clear that the improved approaches to secondary treatment of drinking water discussed above can be effective in removing most of the pharmaceuticals. However, new cost-effective approaches are needed to remove pharmaceutical residues before they reach the aquatic environment, one of which is adsorption.

### 3.2. Adsorption

**Figure 2:**
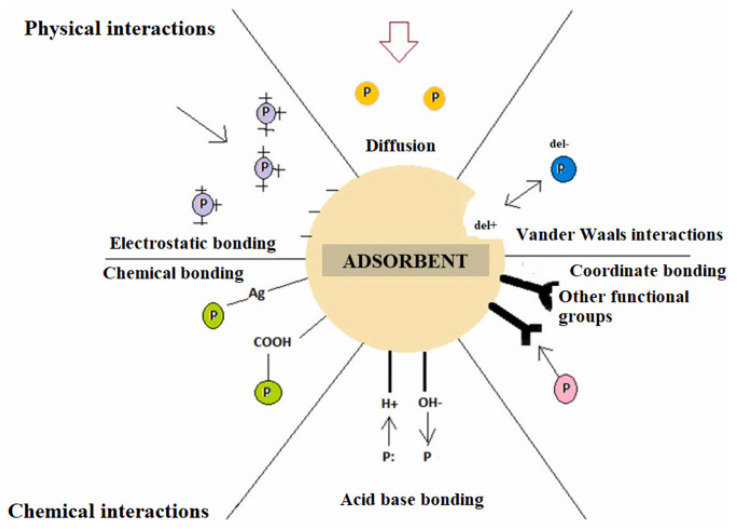
Different types of interactions between adsorbent and adsorbate. Reprinted from [40], with permission from Elsevier.

Adsorption is one of the most effective, versatile, and affordable treatment methods in wastewater treatment plants due to its simplicity and the wide range of organic and inorganic pollutants it removes [38]. It is a mass transfer process in which contaminants (adsorbates) migrate from the liquid phase to the surface of a solid material (adsorbent) through physical and chemical interactions. Adsorption efficiency depends on multiple factors, including adsorbent properties, adsorbate concentration in solution, the presence of accompanying impurities, solution pH, temperature, and contact time. The adsorption process occurs from the solution volume to the adsorbent surface and to the pore surface by diffusion. The adsorption mechanism involves physical and chemical interactions on the adsorbent, including electrostatic attraction, ion exchange, hydrogen bonding, van der Waals interactions, surface complexation, and π-π interactions [39]. Different types of interactions between adsorbent and adsorbate are explained in Figure 2.

The advantage of adsorption technology is its high degree of purification (over 99%). Adsorption material can be used in a wide range of pH and temperature values. It can be regenerated, which extends its usage life. On the other hand, periodic regeneration reduces adsorption capacity, and over time, the adsorbent needs to be replaced.

The most common adsorbents used in wastewater treatment are activated carbon, clays, zeolites, etc. The physical and chemical properties of activated carbon depend on the source and the method of preparation. The adsorption of pharmaceuticals on inexpensive activated carbon decreases with increasing pH and temperature in the range of 4–40 °C. Its large surface area and easy availability, despite its low efficiency, are prerequisites for its frequent use in wastewater treatment. Studies suggest that combining several low-cost adsorbents can improve pharmaceutical removal efficiency [41]. Disadvantages of activated carbon are long adsorption time, poor regeneration, and reduced porosity. As the adsorption capacity of activated carbon decreases, it needs to be replaced, resulting in additional and ongoing costs [42]. In addition, agricultural and industrial wastes such as sludge, fly ash, red mud, etc., are considered as cheap adsorbents. Agricultural and industrial wastes such as sludge, fly ash, red mud, etc., are considered as cheap adsorbents. Nevertheless, the search for more efficient, cheaper, and environmentally friendly materials for scrubbing is still relevant [21,43].

**Figure 3:**
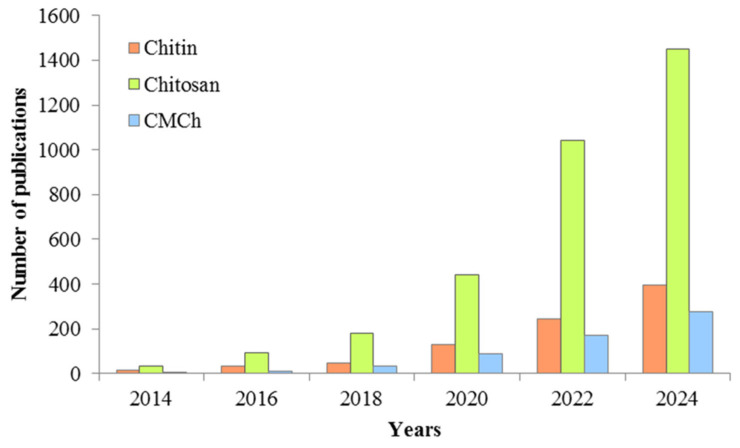
Wastewater treatment from antibiotics using chitin/chitosan/carboxymethyl chitosan by adsorption method.

The choice of adsorbent for pollutant removal depends largely on the concentration and nature of the pollutant present in the water, the adsorption capacity, and its efficiency. Recently, much attention has been paid to adsorption methods using so-called “low-cost wastes”, namely agricultural and industrial wastes, including polysaccharides, for wastewater treatment [3,44,45,46,47,48,49]. According to www.ScienceDirect.com (accessed on 31 March 2025), the number of papers on the use of chitin, carboxymethyl chitosan, and, especially, chitosan for the removal of antibiotics from the aquatic environment, is increasing every year (Figure 3).

## 4. Chitosan-Based Adsorption Materials

**Figure 4:**
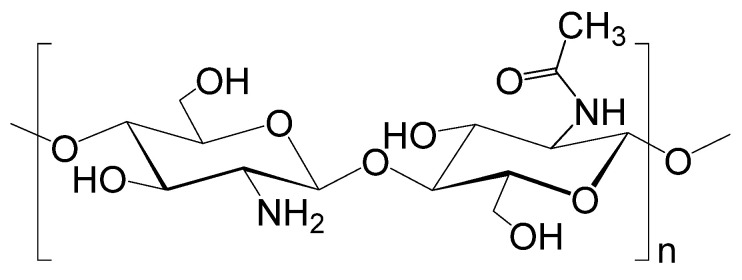
Chemical structure of chitosan—*β*-(1→4)-2-acetamido-D-glucose and *β*-(1→4)-2-amino-D-glucose.

The use of polysaccharides as adsorbents is due to their economic advantages, availability, and renewability of the raw material base. Chitin is considered an affordable and environmentally friendly waste. Chitin serves as a structural element of arthropods in crustaceans (crabs, lobsters, shrimps), in insects (wasps, bees, beetles), in arachnids (spiders, mites, scorpions), and is also found in the cell walls of fungi, algae [50]. Chitosan is a functional derivative of chitin obtained by deacetylation of the acetamide group at the C-2 position of the monomer N-acetyl-D-glucosamine into an amino group, with a degree of deacetylation (DD) of more than 50% [51]. Chitosan is a linear polysaccharide consisting of randomly distributed monomeric units of D-glucosamine and N-acetyl-D-glucosamine linked by a β-(1→4)-glycosidic linkage (Figure 4). Each glucoside residue in the structure contains two hydroxyl groups and one amino group, thanks to which chitosan can be modified according to the task [52]. Chitosan has the ability to remove pollutants from water, such as dyes, agrochemical residues, some pharmaceuticals, metal ions, phenols, various anions, etc. [53].

In aqueous medium (pH ≤ 6.3), the chitosan molecule can interact with negatively charged macromolecules and anions due to its overall positive charge. Based on the properties and applications already known for the biopolymer, the ability of chitosan to adsorb drugs from aqueous media may help to replace expensive and inefficient adsorbents already used in wastewater treatment [53].

The potential use of chitosan as an adsorbent for the removal of pharmaceuticals is influenced by the DD of chitosan. An increase in DD of chitosan leads to the presence of more amino groups, increased tensile strength, and crystallinity. The distribution of acetyl groups along the main chain affects the mechanical properties, swelling, and thermal properties of chitosan. The molecular weight (MW) of chitosan does not significantly affect its ability to adsorb contaminants. The surface area of chitosan-based adsorbent depends on the particle size, as larger particle sizes lead to lower absorption of pollutants [54]. Chitosan particles with size characteristics of less than 100 nm (nanoparticles) have a larger surface area. This makes the functional amino and hydroxyl groups more available for interaction, which is important in the sorption process of drugs. The structure of chitosan nanoparticles depends on their size and shape. Both characteristics influence the adsorption process. However, it should be noted that the use of chitosan nanoparticles has limitations. When sorbed under uncertain pH levels or in acidic environments, such particles are unstable and degrade over time. Additional risks are associated with the long-term stability and potential toxicity of their nanoparticles. Further studies are needed to assess their safety and particle behaviour under optimal conditions during adsorption to optimise their use and minimise potential risks. Nevertheless, chitosan can be functionalised with other substances to improve their specific adsorption and process efficiency [55].

The removal of rifampicin (RIF), streptomycin (STM) and IBU by adsorption on chitosan under different processing parameters was investigated in detail by [56]. The chitosan used in this work in the form of submicroparticles (413–1527 nm) was obtained from the shells of the mud crab *Scylla serrata* (average viscous molecular weight −8.96 kDa, degree of acetylation −9.6%). The specific surface area of the chitosan material was (6.57 ± 0.05 m^2^/g), the mean pore diameter was 87.3 ± 3.1 nm, and the mean pore volume was 0.0397 ± 0.0017 cm^3^/g. These properties are significantly better than those reported for commercially available chitosan (2.10 m^2^/g, 41.58 nm and 0.01977 cm^3^/g). The adsorbent was characterised before and after adsorption by a series of spectroscopic and microscopic analyses. The adsorption mechanism of drugs on chitosan was interpreted on the basis of surface charge, surface area, pore size, surface morphology, vibrational spectra, crystal structure, and thermal properties. The authors suggest that hydroxyl and amino groups on the chitosan surface are hydrogen donors. They bind to oxygen atoms of the heterocyclic ring and carbonyl groups of RIF, STM, and IBU, which are hydrogen acceptors. The maximum adsorption capacities of RIF, STM, and IBU on chitosan were 66.91, 11.00 and 24.21 mg/g, respectively. These values are higher compared to the maximum adsorption capacity on various agricultural wastes. The authors believe that the chitosan used is a practical and economical adsorbent for the removal of pharmaceutical compounds from wastewater.

The possibility of removing furosemide from aqueous solution by an adsorption process on chitosan films as a safe, stable and recyclable adsorbent was investigated [57]. Furosemide, a drug actively recommended by health professionals, is eventually detected in a wide range of concentrations as a contaminant in the aquatic environment. The drug is one of the most dangerous environmental pollutants [1]. The toxicological effect of furosemide is due to the formation of toxic metabolites, and 40–69% of it is excreted in its original form. Commercial chitosan powder (from crab shells, high viscosity, DD less than 75%) was used to form films. The influence of several experimental parameters, such as pH values, ionic strength, amount of adsorbent/pollutant, and temperature values on the adsorption process was investigated. Physical adsorption of furosemide occurred on the heterogeneous surface of chitosan. Most of the contaminant (90%) was adsorbed in 2 h; the adsorption capacity was 3.5 mg/g. The desorption of furosemide from chitosan films was 100% using concentrated NaCl solution (1.5 M). This means that there is a possibility of multiple uses of the adsorbent [57].

It follows from the above that chitosan is an available, cost-effective adsorbent for the removal of some pharmaceuticals. However, there are difficulties with its practical application, such as non-selective binding on the adsorbent surface, reduced mechanical strength, relatively low specific surface area, low porosity, sensitivity to pH changes, etc. [45].

## 5. Adsorbents Based on Modified Chitosan

The presence of amino and hydroxyl groups in the chitosan molecule provides opportunities for its chemical modification. This procedure is most often aimed at increasing the solubility of the polymer. Modification is also designed to eliminate the key disadvantages of chitosan: to increase the specific surface area of the material and enhance its hydrophilicity. In the context of adsorption, as the most promising purification method, modified forms of chitosan and its composites are expected to acquire key properties for sorption: high surface area, increased mechanical strength, and a large number of functional groups for effective binding of pollutants. There are several ways to modify chitosan in order to obtain adsorbents with improved properties. These include monomer grafting followed by polymerisation, physical or chemical cross-linking, obtaining chitosan derivatives, and composites, including those with inorganic substances, etc. [3].

Examples for the preparation of chitosan-based adsorbents for the removal of some antibiotics are discussed below (Table 2).

### 5.1. Chitosan-Based Adsorbents with Grafted Monomers

Granular hydrogel with a three-dimensional structured network was used as an adsorbent for the removal of two typical fluoroquinolone antibiotics, CIP and enrofloxacin (ENR) [58]. The adsorbent was based on chitosan with a DD of 85% and an average MW of 3.0 × 10^5^ Da. An acrylic acid monomer (CTS-PAA) was grafted to it by radical polymerisation in solution. The grafting of new functional groups, including carboxyl groups, onto the main chain of the chitosan molecule should help to increase the adsorption points and improve the selectivity of the adsorbent. The adsorption capacity of the hydrogel was investigated at initial antibiotic concentrations ranging from 10 to 600 mg/L, in the pH range 2.0–9.0. The presence of numerous ionised carboxyl groups in the hydrogel contributed to the maximum adsorption at pH 3.0 and a higher adsorption capacity of 267.7 mg/g for CIP and 387.7 mg/g for ENR. The researchers evaluated the reusability of the adsorbent. Five adsorption–desorption cycles resulted in an adsorption percentage of more than 85%. 

The problems of improving the textural properties of chitosan, including low porosity, reduced surface area, sensitivity to pH changes, and difficulty in recovery post-course of treatment, were solved by combining chitosan with polyaniline (PANI) [70]. PANI is considered a good adsorbent for pollutants. Chitosan-based adsorbents (CS) with an average MW, obtained with polyaniline (PANI/CS), were used to remove acetaminophen (AAP) from an aqueous solution. Analysis of the adsorbent surface area demonstrated that the presence of PANI in the PANI/CS increased its surface area and porosity. Functional groups such as imine and amine in polyaniline can effectively react with various pollutant molecules in water. The effects of pH, temperature, adsorption equilibrium time and initial concentration of AAP on the adsorption process were investigated in this work. It was shown that the maximum adsorption capacity at 25 °C and optimum pH 7.0 was 385.25 mg/g, 359.84 mg/g and 242.49 mg/g for PANI/CS, CS, and PANI, respectively. The adsorption is mainly attributed to hydrogen bonding, π-π interactions, pore filling and chemisorption as a possible mechanism for AAP removal by PANI/CS. When reused, the PANI/CS adsorbent retained approximately 69% of its original capacity after five adsorption cycles. The authors believe that the adsorbent can be used for the adsorption of AAP from wastewater due to its good selectivity and adsorption capacity [70].

The synthesis of a new grafted copolymer, in the form of a hydrogel, derived from chitosan, itaconic acid (ITA), and by free radical copolymerization with acrylamide (AM), was proposed in the study [71]. The main chain of the polymer contains several branches made up of polymer chains with different chemical compositions. This type of copolymer can modify the chemical structure of the polymers, which contributes to the improvement in adsorption properties [58]. ITA, an unsaturated dicarboxylic acid, has a wide range of applications, including the preparation of superadsorbents. The polymer hydrogel (chitosan-g-P (ITA-co-AM)) was used for the adsorption of metformin (MET), one of the most commonly used antidiabetic drugs, from aqueous solution. Factors affecting adsorption, such as adsorbent dose, contact time, concentration and pH of the solution, were investigated. The maximum adsorption of MET was 89.44% (20 mg/g, pH = 7.0, 60 min, at 25 °C). After six cycles of adsorption and desorption, it was found that the chitosan-g-P (ITA-co-AM) had an adsorption efficiency of 70.88%. The authors believe that the synthesised hydrogel is suitable for the removal of toxic drug impurities.

### 5.2. Adsorbents Based on Chitosan Derivatives

An adsorbent based on carboxymethyl chitosan (CMC) with Na-montmorillonite (Na-Mt) (wt%, SiO_2_ 57.12, Al_2_O_3_ 14.98, Fe_2_O_3_ 5.89, MgO 4.96, CaO 3.34, Na_2_O 2.61, K_2_O 0.38, TiO_2_ 0.83) was obtained and studied in [72]. The new material was used to study the adsorption of tetracycline and chlortetracycline at various adsorbent concentrations, pH, temperature, and contact times. The dependence of adsorption efficiency on pH was demonstrated. Electrostatic interaction between negatively charged antibiotic anions and positively charged CMC promoted adsorption at pH values between 4 and 7. The initial antibiotic concentration was 100 mg/L, and the adsorption capacity was 48.10 mg/g. The removal of tetracycline and chlortetracycline quickly reached equilibrium within 2 h of contact time. At equilibrium adsorption, the observed removal rates of tetracycline and chlortetracycline were 95.21% and 96.36%, respectively, at a CMC-Mt dosage of 2 g/L.

Tetracycline in its zwitterionic form (zTC) was also selected as the target antibiotic in [73]. The authors used chitosan (CTS, DD > 75%; MW 50–190 kDa; η 20–300 cP for 1 wt.% in 1% acetic acid at 25 °C) and chitosan functionalized with either 1-naphthaldehyde (RCN) or 2-hydroxy-1-naphthaldehyde (RCN1). The real effectiveness was evaluated in adsorption experiments, electronic structure calculations, and molecular dynamics (MD) simulations. They were used to study the adsorption of the antibiotic on monomers of CTS, RCN, and RCN1, and provide molecular-level insight about the relevant interactions. The best conditions for tetracycline adsorption were achieved at pH = 6 and solid–liquid ratio = 5 mg/mL. Under these conditions, the maximum adsorption capacity (qm) of RCN1 (qm = 1.24 mg/mL, adsorption efficiency < 51.7%) was three times higher than that of CTS (qm = 0.41 mg/mL, adsorption efficiency < 13.1%). According to the electronic structure calculation and MD simulation, the authors concluded that tetracycline adsorption is more preferable on RCN1 than on RCN and CTS, due to the greater stability of the zTC–RCN1 complex.

A nanofiber membrane based on N,O-carboxymethyl chitosan (N,O-CMCS) and polyethylene oxide (PEO) with MW 900,000 g/mol was prepared by electrospinning. The N,O-CMCS derivative was synthesised on the basis of low molecular weight chitosan with MW 50,000–190,000 g/mol, DD 75–85%. Electrospinning solutions with different weight ratios (*w*/*w*) of N, O-CMCS/PEO were used in the experiments. A ratio of 4:3 (*w*/*w*) was found to be optimal. Electrospinning was performed at room temperature and relative humidity in the range of 16% to 20%. The best fibre formation (176 ± 40 nm) was obtained under these experimental conditions. It was observed that nanofibre formation was strongly influenced by humidity, because of the more stable fibre jet obtained during winter and summer seasons (taking into account humidity fluctuations). The optimum conditions for nanofibre stabilisation were 140 °C for 30 min. Under these conditions, the fibres were strengthened without any surface modification, and the membranes became stiffer. Sorption capacity tests were performed using high-performance liquid chromatography and ultraviolet diode detector (HPLC-UV DAD) under experimental conditions of controlled pH. The developed membranes showed excellent ability to remove FLX from water at pH 8.0 (adsorption capacity up to 79.7 ± 7.9 mg/g). Based on the experimental results obtained and the comparison with other existing sorbents, the authors suggested that N,O-CMCS-PEO nanofibres are effective and suitable for the removal of pharmaceutical residues such as FLX from water [74].

N-succinyl chitosan (NSCS), a water-soluble derivative, was used with the intention of finding out whether hydrophobic moieties such as -CH_2_-CH_2_- would increase the adsorption capacity of the tested sample [75]. The authors built on the results of a previous article [74], on the adsorption of FLX on a nanofibre membrane composed of N,O-carboxymethyl chitosan (N,O-CMCS) and PEO. It is possible that weak intermolecular hydrogen bonding due to -NH-CO- and -OH groups along the macromolecular chains as a result of NSCS synthesis, hydrophobic interactions between hydrophobic -CH_2_-CH_2_- fragments, acetyl groups and glycoside rings affect the adsorption capacity of the nanofibre membrane composed of N-succinyl chitosan and polyethylene oxide (NSCS/PEO). A nanofibre membrane based on NSCS and PEO with a MW of 900,000 g/mol was prepared by electrospinning. The N-succinyl derivative was synthesised based on low molecular weight chitosan with a molecular weight of 50,000–190,000 g/mol, DD 75–85%. Electrospinning solutions prepared with different mass ratios of NSCS to PEO—3:2, 5:5 and 6:4—were used in the experiments. A ratio of 6:4 was found to be optimal. Electrospinning was performed at room temperature and a relative humidity of 30–40%. The obtained NSCS/PEO nanofibres had an average diameter of 183 ± 38 nm. The optimum adsorption conditions of FLX on NSCS/PEO nanofibres were at pH 8.0. The maximum adsorption capacity of FLX on NSCS/PEO was 82.3 ± 3.4 mg/g. According to the results, FLX can be desorbed from the NSCS/PEO nanofibre membrane and reused up to four times without significant loss of adsorption capacity. The authors believe that the present study extends the possibilities of modifying chitosan by increasing its content of hydrocarbon moieties, which affect the adsorption capacity, taking into account the presence of hydrophobic moieties in their structures. During the adsorption process on the nanofibre membrane, hydrophobic and electrostatic interactions take place with the target molecule.

**Figure 5:**
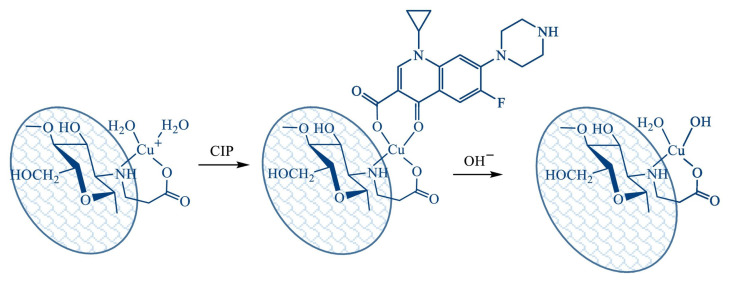
Possible schematic illustration of CIP adsorption on Cu(II)-CEC cryogel. Reprinted from [76] with permission from Elsevier.

In their article [76], the authors propose using a metal chelation approach to remove fluoroquinolones from aqueous solutions. It is based on the ability of fluoroquinolones to bind divalent and trivalent metal ions. The study used chitosan with MW = 2.5 × 10^5^ Da and DA = 0.16, from which N-(2-carboxyethyl)chitosan (CEC) with a degree of substitution of 1.06 was synthesised. On its base, a supermacroporous cryogel was obtained by cross-linking with hexamethylene diisocyanate, on which metal ions were further immobilised by adsorption from 0.004 M solutions of FeCl_3_, Cu(NO_3_)_2_, and Al(NO_3_)_3_. Cryogel beads with and without metals were used to bind ciprofloxacin (CIP). Application of initial CEC cryogel for CIP uptake was limited despite relatively high sorption capacity, low affinity, due to strong pH dependence, and low recovery efficacy in the pH range relevant for drinking water treatment (pH 6.5–8.5). The CIP recovery on Cu(II) and Al(III)-chelated CEC cryogels was more than 90% (pH 7–10), with the maximum efficacy reaching 98% for Al(III)-chelated cryogel, while the efficiency of the primary cryogel under the same conditions was 15–18%. A possible schematic illustration of CIP adsorption on Cu(II)-chelated CEC cryogel is presented in Figure 5. The authors suggest that antibiotic adsorption will occur due to complexation on the surface of metal-chelated sorbents. The monomer unit of CEC is a bidentate chelating ligand which binds Cu(II) ions via the nitrogen atom of the amino group and the oxygen atom of the carboxylic group. Vacant coordination positions on the metal centre can be occupied by CIP. Presumably, the Cu(II) ion immobilised in the CEC cryogel coordinates CIP via the oxygen atoms of the deprotonated carboxyl and carbonyl groups, since complexes of this stoichiometry and structure are typical for fluoroquinolones in neutral and alkaline media. The sorption capacity of Cu(II)-CEC cryogel increased with increasing copper content up to 55 mg/g and reached a value of 280 mg/g, corresponding to a Cu(II)/CIP stoichiometry of 1:1. The efficiency of CIP removal from a solution with a concentration of 50 μg/L was 86 ± 2%.

### 5.3. Adsorbents Based on Chitosan Composites

The most commonly described way of modifying chitosan in articles is to form composites and use them as adsorbents for drug extraction from aqueous solutions [54]. Chitosan combined with other organic and inorganic materials, i.e., in the form of composites, shows improved adsorption capacity for the removal of contaminants, including antibiotics. 

TC and CIP are among the most commonly used antibiotics. They are known as broad-spectrum and low-cost drugs. Both drugs are widely used in high doses for treatment. TC is excreted from the body in an unchanged form and in high concentrations. Due to its good solubility in water, TC is found in high concentrations in the surrounding aquatic environment. Therefore, a large number of research groups are working on solving the problem of the adsorption of tetracycline and ciprofloxacin from the aqueous medium. In particular, various composites with chitosan are being studied for this purpose (Table 3).

#### 5.3.1. Adsorbents Based on Composites of Chitosan with Silica

The study [84] presents the development of a silica/chitosan (MW 150 ± 3 kDa, DD 85% ± 0.02) adsorbent modified with glutaraldehyde, which was examined for its ability to remove diclofenac sodium. The authors declare that the sol–gel method using silica precursors is a promising method for producing chitosan-based adsorbent composites. The synthesis process led to an increase in the surface area of chitosan and chemical changes in its structure. A high adsorption capacity of 237.8 mg/g was detected, and a pH of 6.7.

Silica-chitosan nanocomposites were synthesised using the sol–gel method [85]. Researchers used chitosan and silica in mass ratios of 1.25% (*w*/*w*), 2.5% (*w*/*w*) and 5% (*w*/*w*). The 2.5% *w*/*w* chitosan-silica aerogel composition had the largest surface area. The nanocomposite material had a large surface area S_BET_ −343 m^2^/g, a total pore volume of 0.581 cc/g and a pore diameter of 34 nm. The surface area analysis was performed using a method of mathematical description of physical adsorption based on the theory of polymolecular (multilayer) adsorption—the BET method. Due to its structure, the silicon-chitosan nanocomposite material had the ability to adsorb materials containing groups of compounds with both negative and positive charges. The adsorption capacity of the nanocomposite material was tested for the removal of diclofenac, and IBU and CBZ from synthetic seawater at pH 5.7 and 8.5. The highest removal was observed at pH 8.5, in the following order: CBZ > IBU > diclofenac. Among these three pharmaceutical compounds, CBZ was removed most efficiently (89.3%) from the artificial seawater. The adsorption isotherms were consistent with the Langmuir and Freundlich isotherm models, and the adsorption kinetic results were in good agreement with the pseudo-second-order kinetic model of silica-chitosan nanocomposite for all pharmaceutical compounds. The development of this type of environmentally friendly adsorbent material is important for the removal of micropollutants from aquatic environments.

Chitosan/fibrous silica KCC-1 composite was proposed in the paper [86] as a potential environmentally friendly adsorbent for the removal of diclofenac from water sources. Modification of KCC-1 fibrous silica with chitosan was carried out using ultrasound-assisted impregnation at a chitosan-silica ratio of 1:10. The presence of mesopores in the composite increased the availability of diclofenac molecules, thereby increasing the adsorption of diclofenac. The chitosan/fibrous silica KCC-1 composite has an adsorption capacity of 142.01 mg/g, while fibrous silica KCC-1 has a lower adsorption capacity (65.33 mg/g) at an optimal contact time of 40 min, a pH value of 4.0, and an initial diclofenac concentration of 160 mg/L. Adsorption mechanisms include electrostatic interactions, hydrogen bonds, and hydrophobic interactions between the chitosan/fibrous silica KCC-1 composite and diclofenac. However, electrostatic interactions between the cationic functional group (-NH_3_^+^) of the composite and the anionic group of diclofenac (-COO^−^) prevailed.

In summary, chitosan-silicon composites combine the advantages of both materials for effective sorption of pharmaceutical contaminants. They have a developed surface with the ability to control pore size, as well as numerous active centres for effective binding of pharmaceutical molecules. In addition, such composite materials will be safer and more biocompatible compared to synthetic sorbents. The main disadvantages limiting the widespread use of chitosan-silica adsorbents are related to their chemical and mechanical stability under real operating conditions, as well as the complexity of scaling and cost.

#### 5.3.2. Adsorbents Based on Composites of Chitosan with Metals, Metal Oxides

**Figure 6:**
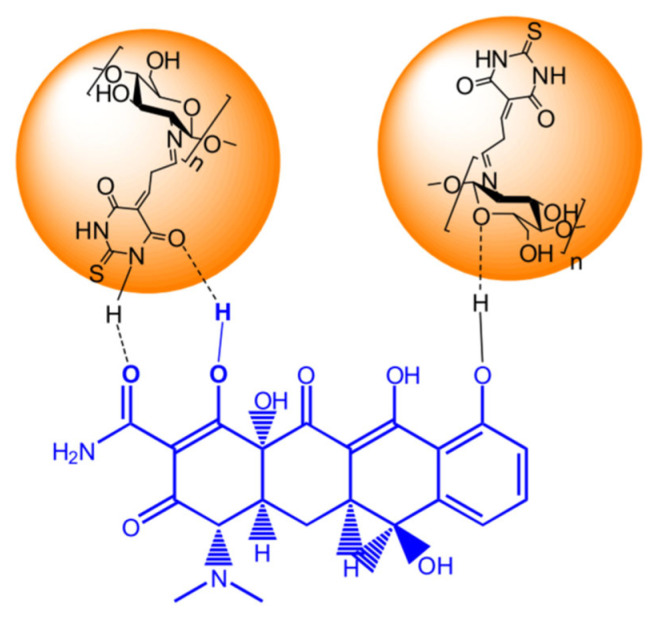
Adsorption mechanism of TC over CTM@Fe_3_O_4_. Reprinted from [77], with permission from Elsevier.

Chitosan in composite form exhibited a higher adsorption capacity to remove pollutants in [77]. A nanocomposite (CTM/Fe_3_O_4_) was prepared by polycondensation reaction between chitosan (MW of 190–310 kDa, DD of 75–85%), thiobarbituric acid, and malonic dialdehyde in an acidic medium, followed by incorporation of Fe_3_O_4_ nanoparticles. The nanocomposite was used for the extraction of tetracycline (TC) from aqueous solution. The adsorption process of TC on the composite was optimised by several factors such as pH, concentration, contact time and temperature. The highest adsorption capacity of 215.31 mg/g was achieved under such optimum conditions as adsorbent dosage of 0.05 g, TC concentration of 60 mg/L. A possible mechanism of surface adsorption of TC occurs due to the π-π and hydrogen bonding interaction (Figure 6).

After adsorption of TC, the nanocomposite was dispersed in 40% methanol solution, washed several times, and the regenerated nanocomposite was used again for adsorption. After six cycles, the adsorption capacity of the nanocomposite was 203.41 mg/g. The results showed that the nanocomposite is an adsorption material with superparamagnetic properties. This enables both separation and extraction of the adsorbent from the aqueous solution using an external magnet. The high adsorption capacity of the nanocomposite may be due to a more developed structure of the composite adsorbent with magnetic particles, either due to improved pore properties or enrichment with active functional groups. Thus, the nanocomposite may find application as an effective and inexpensive adsorbent for water purification of antibiotics. 

An adsorbent with magnetic properties (CS/Fe_3_O_4_) based on chitosan (CS) and iron salt FeCl_2_ × 4H_2_O, which was used to remove TC from aqueous solution, was obtained in [78]. The magnetic adsorbent was synthesised by the co-precipitation method. From the analysis results, CS/Fe_3_O_4_ was found to have a crystalline and mesoporous structure, uneven surface area, and ferromagnetic properties. It was observed that the adsorbent efficiency was influenced by initial TC concentration, adsorbent dosage, pH, and ionic strength. The maximum adsorption capacity was determined at pH 7.0 and was 211.21 mg/g. The equilibrium and kinetic studies were described using Sips and Elovich models, which suggest that the adsorption process occurs on heterogeneous surfaces by a chemisorption mechanism. The CS/Fe_3_O_4_ adsorbent was shown to be effective in the removal of TC after three adsorption/desorption cycles. After five cycles, its sorption capacity decreased by 15–18%.

A composite based on chitosan (medium molecular weight, 75–85% deacetylated) combined with an organometallic framework (SFMOF/BM) that was additionally surface modified with various amounts of 3-aminopropyltrimethoxysilane was obtained in [59]. The iron-based framework attracted attention because of its chemical stability and non-toxicity. The synthesis was carried out by mixing a suspension of chitosan in ethyl alcohol with the surface-modified framework in a ratio of 1:2 *w*/*w* under stirring for 4 h to form a composite. The three-dimensional organometallic framework is a class of novel crystalline and hybrid porous materials linked by transition metals (clusters) to bridging organic ligands. Its introduction into the composite allowed for increasing the surface area of the adsorbent with well-defined, controlled pore size [87]. The composite, with a functionalized surface, was used as an adsorbent for the removal of contaminants, including antibiotics TC and doxycycline. The adsorption experiments were carried out under optimum pH 5.40. The adsorption capacity for TC and doxycycline was 388 and 264 mg/g, respectively. After five cycles of using the composite adsorbent, the adsorption efficiency was more than 70%. The authors believe that the developed composite adsorbent, due to its high adsorption capacity and easy regeneration, can be considered promising for water treatment to remove organic pollutants.

The synthesis of magnetic nanocomposites in the presence of chitosan, copper chloride, cobalt chloride, and iron chloride III salts is described for the first time [79]. A defined stoichiometric ratio (0.5:0.5:2) of the salts was mixed with chitosan after dissolution in distilled water. The preparation of this magnetic nanoadsorbent involves a fast, environmentally friendly, and highly efficient microwave synthesis without the use of toxic solvents and surfactants. The obtained CuCoFe_2_O_4_/Ch magnetic nanocomposite was used to remove the antibiotic TC from aqueous media. The best removal efficiency of TC was 93.07% under optimum conditions (initial antibiotic concentration 5 mg/L, pH 3.50, adsorbent dosage 0.4 g/L, and temperature 25 °C). Under the same conditions, the magnetic nanocomposite CuCoFe_2_O_4_/Ch adsorbed 67% of TC from real wastewater. It was shown that the magnetic nanocomposite CuCoFe_2_O_4_/Ch has a high adsorption potential for TC from both synthetic and real wastewater. After four adsorption–desorption cycles, the adsorbent showed a removal efficiency of about 82.16%. The authors believe that some advantages of this study, i.e., the rapid and environmentally friendly method of adsorbent synthesis, its kinetic and magnetic properties, serve as a prospect for the application of this adsorbent. 

**Figure 7:**
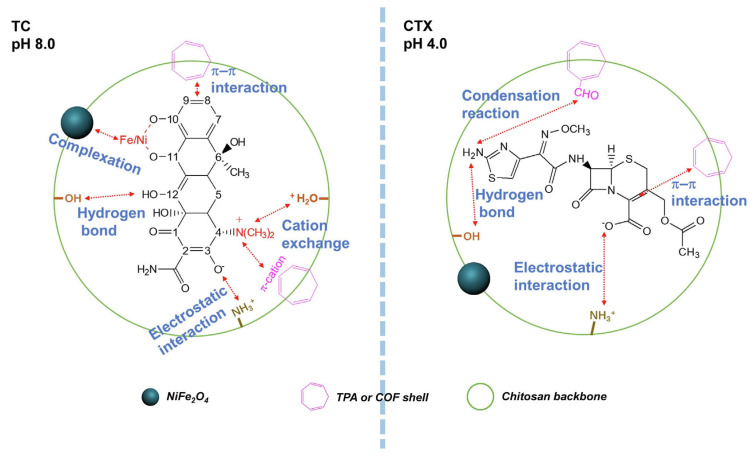
Adsorption schematic diagram of TC and CTX on NCCT. Reprinted from [69], with permission from Elsevier.

Li et al. synthesised NiFe_2_O_4_-COF-Chitosan magnetic nanocomposites that were cross-linked with terephthalic aldehyde films (NCCT) [69]. Chitosan with MW = 200,000 g/mol, DD 95% was used in the work. NCCTs were used to remove TC and cefotaxime (CTX) from aqueous media. The NCCT film had a rough surface and an average thickness of 7.5 ± 0.5 µm. The kinetics of antibiotic adsorption corresponded to the pseudo-second-order model, and the isotherm of adsorption corresponded to the Freundlich model. The maximum efficiency of TC removal was 93.07% at pH 3.50, 25 °C, while the initial concentration of the antibiotic was 5 mg/L, and the dosage of the adsorbent was 0.4 g/L. The maximum removal of TC and CTX was 388.52 mg/g at pH 8.0 and 309.26 mg/g at pH 4.0, respectively. The authors proposed reaction mechanisms for both antibiotics. The main mechanism of TC adsorption on NCCT consisted of complexation, cation exchange, electrostatic attraction, formation of hydrogen bonds, and π–π interaction. The mechanism of CTX adsorption, in addition to electrostatic attraction, the formation of hydrogen bonds, and the π–π interaction, also includes a condensation reaction (Figure 7). In addition, good regeneration ability was shown, so after four cycles of adsorption–desorption, the efficiency of adsorbent removal was about 82.16%.

Fe_3_O_4_-chitosan nanoadsorbent (CTS-MNP) was synthesised in [60]. Chitosan with MW 111 kDa and DD 80–85% was used. The nanoadsorbent was used to remove metronidazole (MTZ), an antibiotic from the nitroimidazole family, which is widely used to treat infectious diseases caused by bacteria and protozoa. The researchers applied R software using response surface methodology to investigate the effect of the composition of the input factors and the output response. The predicted optimum conditions considering the maximum removal efficiency (100%) were calculated for the second-order model, and the following data were fixed (pH 3.0; CTS-MNP dosage, 2 g/L, contact time, 90 min, and MTZ concentration, 10 mg/L). The experimental results for the response were found to be in good agreement with the model predictions. The maximum adsorption of the antibiotic was 97 mg/g. Thermodynamic studies showed that the adsorption of MTZ on CTS-MNP is spontaneous and endothermic. The authors believe that the CTS-MNP nanoadsorbent can be used to treat industrial and hospital wastewater from MTZ.

Researchers proposed and obtained a new magnetic biocomposite adsorbent called AgZnFe_2_O_4_/Ch, which was used to remove MTZ from water [61]. Previously, a Fe_3_O_4_-chitosan nanoadsorbent was described for the removal of the same antibiotic [60]. Proper surface coating is important to increase the adsorption capacity of the adsorbent. The advantages of AgZnFe_2_O_4_/Ch adsorbent, according to the researchers, include the rapid, environmentally friendly, and highly efficient production of this magnetic nanoadsorbent with chitosan, which is obtained without the use of harmful solvents and surfactants. Metal chlorides Fe^3+^/Zn^2+^/Ag^1+^ in a stoichiometric ratio of 1:0.5:0.5 were used in the synthesis. The addition of chitosan favoured the preservation of the AgZnFe_2_O_4_ crystal structure and surface stability. A pH of 7, a contact time of 50 min, a temperature of 25 °C, an adsorbent dosage of 0.9 g/L, and an initial MTZ concentration of 10 mg/L were found to be the most suitable for sorption. The effective adsorption under optimum conditions was 65.53% (7.28 mg/g). The authors believe that, based on this study, MTZ can be effectively removed from wastewater using the proposed magnetic biocomposite adsorbent AgZnFe_2_O_4_/Ch.

#### 5.3.3. Chitosan-Based Adsorbents with Carbonaceous Materials

Particles ≤ 5 mm of dried hydrogel, based on CS from shrimp shells, with a DD of 82.5 ± 1.2% and biochar (BC) from rice husk (size 0.5 ± 0.01 mm) were used as adsorbent (Chitosan/BC) to remove the antibiotics CIP and ENR from aqueous solution by the adsorption method [62]. The Taguchi method was used to determine the optimum adsorption conditions for specific antibiotics [88]. The optimum conditions for adsorption of CIP and ENR on the composite are 1.0 and 1.5 g/L for 300 and 450 min, respectively, at pH = 7.0. The adsorption isotherms for these antibiotics follow the Langmuir isotherm with a maximum adsorption capacity of 106.038 mg/g for CIP and 100.433 mg/g for ENR. The authors suggest that the adsorption mechanism involves π-π electron donor–acceptor interactions, hydrogen bonding, and electrostatic attraction. A total of 97.4% of CIP and 90.2% of ENR were removed. It is non-toxic to *Chlorella* sp., confirming its potentially safe application for actual wastewater treatment. The mortality rate of *Chlorella* sp. using the resulting hydrogel (72 h) was 7.8 ± 0.23% for CIP and 37.15 ± 2.71% for ENR, confirming its potentially safe application for actual wastewater treatment.

**Figure 8:**
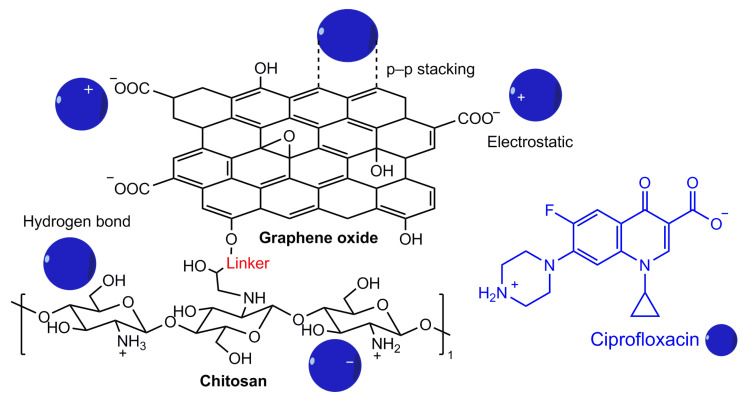
Adsorption mechanisms between chitosan-graphene oxide and antibiotic ciprofloxacin.

According to several authors, the incorporation of carbonaceous materials into the structure of chitin/chitosan is an effective way to improve its mechanical and thermochemical properties [89]. Carbonaceous materials can enhance the adsorption capacity of biopolymers by increasing their functionality and modifying their pore properties. The most commonly used carbonaceous materials are graphene oxide (44%) and activated carbon (24%), followed by carbon nanotubes (19%), biochar (7%), and graphene (6%). The application of chitosan/chitin-carbon composites for the adsorption of various water pollutants, including some antibiotics such as CIP and TC, which are widely used to treat bacterial infections, has been studied. Their presence in water can lead to the emergence of resistant bacteria, which is a potential threat to human and animal health. The chitosan-graphene oxide and chitosan-activated carbon composites showed high adsorption capacity towards antibiotics, which amounted to 495.64 mg/mL for TC and 526.31 mg/mL for amoxicillin. And the chitosan-biochar composite had an adsorption capacity of more than 76 mg/mL for CIP [63,65,81]. High adsorption capacity was exhibited by (magnetic) chitosan-graphene oxide towards CIP (282.9 mg/mL), whereas the adsorption capacity for chitin-graphene oxide towards the same antibiotic was 73.0 mg/mL [82,83]. This is probably due to the structure of chitosan, the appearance of a free primary amino group, the increase in surface area, porosity, or due to the magnetic properties of the chitosan-graphene oxide (Fe_3_O_4_) composite. A possible mechanism of adsorption between an adsorbent based on graphene oxide and chitosan with the antibiotic ciprofloxacin is shown in Figure 8. Mechanism involves π-π electron donor–acceptor interactions, hydrogen bonding, and electrostatic attraction. Thus, carbonaceous materials play a significant role in enhancing the efficiency of chitosan towards micropollutants. In addition, the biopolymer composites with magnetic material are more stable, convenient for separation, and recyclable.

Aerogel microspheres based on graphene oxide/chitosan (GO/CS) were used for levofloxacin (LOF) adsorption. Chitosan with DD 95% was used in the work. Aerogel microspheres were synthesised by emulsification and cross-linking, followed by freeze drying. The dependence of the GO content on the adsorption capacity is shown, so with the addition of 40 wt.%, it increased from 9.9 to 45.6 mg/g. The adsorption efficiency was also influenced by pH and temperature values; it was shown that at pH = 8 and T = 25 °C, it was 51.5 mg/g. The authors investigated the thermodynamics and kinetics of this process using four different LOF adsorption models. It was found that LOF adsorption is exothermic and generally corresponds to the law of velocity of the pseudo-first order [68]. 

The results obtained in [90], showed that a multifunctional and stable composite material (GO-AC-CS) consisting of graphene oxide (GO), activated carbon (AC), and chitosan (CS, flakes, DD 80%) exhibited enhanced structural stability. The structural characterisation by powder X-ray diffraction indicated the incorporation of activated carbon and chitosan into GO. The surface area of GO-AC-CS was 214 m^2^/g, higher than that of GO (132 m^2^/g). The particle sizes of GO and GO-AC-CS composite were 237 and 336 nm, respectively. The removal efficiency of pharmaceutical contaminants using the GO-AC-CS composite was found to be 90.7% and 89.8% for acetaminophen (ACP) and CBZ, respectively. The Langmuir isotherm model was found to be the best fit and estimated maximum adsorption capacities of 13.7 and 11.2 mg/g for ACP and CBZ, respectively. Regeneration experiments showed that 80% removal of the adsorbed substance could be achieved with the appropriate organic solvent. ACP desorption was significantly higher in the polar solvent (70–80%), whereas CBZ desorption was better in the non-polar solvent (60%). The overall results showed that the obtained multifunctional and stable GO-AC-CS is a promising adsorbent for the removal of pharmaceutical and personal care pollutants from wastewater and can act as a good tertiary treatment option in wastewater treatment systems.

Three-dimensional aerogels, based on chitosan and gelatin with the incorporation of graphene oxide (GCGO), either in the total gel volume or by application on the surface, have been analysed as adsorbents for water purification [64]. Low molecular weight chitosan was used to form the aerogel. In order to select the best adsorbent structure, the adsorption capacity was studied, including on two fluoroquinolone antibiotics, ofloxacin and CIP. For both types of 3D aerogels, the adsorption capacity of the tested antibiotics differed insignificantly and was 5–8 mg/g. Control aerogels consisting of chitosan and gelatin (30:70 *w*/*w*), without the addition of graphene oxide, obtained by the casting technique, showed no adsorption. Apparently, the presence of a certain amount of graphene oxide in the aerogel is a key factor for the removal efficiency of antibiotics from aqueous solutions. In the studies, a limited amount of graphene oxide (2% *w*/*w*) was used in the preparation of two types of graphene oxide aerogels. The authors believe that the developed fabrication process allows the use of a higher amount of graphene oxide. This will increase the adsorption capacity, which means that 3D aerogels can be used for drinking water purification. Graphene oxide is one of the most promising materials for creating new and effective adsorbents.

A sponge with multiple active groups -OH, -COOH, -NH_2,_ and -SO_3_H was prepared from carboxymethyl cellulose (CMC) and carboxyalkyl chitosan (GCC) cross-linked with genipin, with the addition of sulfated graphene oxide (CMC/SGO-GCC). It was used as an adsorption material for sulfanilamide antibiotics, namely, sulfamethoxazole (SMX) and SPD, from aqueous solutions [91]. SMX and SPD are actively used in livestock and aquaculture due to their broad-spectrum antibacterial properties. At the same time, they negatively affect the metabolism of fish and have toxic effects on algae [92]. CMC/SGO-GCC hydrogel was obtained by ultrasonic treatment for 40 min followed by heating at 60 °C for 5 h by ultrasonic dispersion. The maximum adsorption capacity of SMX and SPD on the adsorbent was 312.28 and 161.89 mg/g, respectively, at 298 K. It was observed that the adsorption capacity of the antibiotics was high within 10 min, equilibrium was reached after 30 min, and the sorption rate remained almost constant. The reuse experiments showed that CMC/SGO-GCC retained high adsorption capacity for SMX and SPD after reuse. This study shows that CMC/SGO-GCC is a promising material for the adsorption of SMX and SPD.

A composite material (Ch/Fe_3_O_4_/rGO) formed from magnetic nanoparticles and reduced graphene oxide (rGO) combined with low molecular weight chitosan (Ch) was obtained in [66]. The authors investigated the possibility of using the composite as an adsorbent to remove cefixime from aqueous solutions. Cefixime is a broad-spectrum antibiotic that inhibits bacterial cell wall synthesis. The best experimental results were obtained at pH 8.0 when the antibiotic concentration was 50 mg/L, the amount of adsorbent was 5 mg, and the adsorption capacity was 29.99 mg/g. The authors note that the research is the first to develop a sorption process on a sorbent based on composite magnetic nanoparticle, reduced graphene, and chitosan granules for the extraction of real industrial waste.

Graphene-based materials, such as graphene oxide and reduced graphene oxide, expand the range of possibilities for composite formation when introduced into polymer matrices. This type of material is relevant in the formation of multifunctional and stable composites to improve the dispersibility of the graphene-based material in the polymer matrix. The advantage of the GO and rGO structure is the presence of oxygen-containing groups. Depending on the oxidation conditions, these are carboxyl (C=O), hydroxyl (C-OH), and epoxy (C-O) groups, which make the surface hydrophilic and reduce the interlayer forces on the surface. The existing large-scale production of graphene and the diversity of graphene materials with different morphologies and properties imply an expansion of its optimal applications. However, the environmental safety of materials, including chitosan, containing oxidised or reduced forms of graphene, is still poorly understood. At present, there is little data available to assess their toxic effects on aquatic biota [93].

Thus, this section discussed the potential of modified chitosan-based materials as effective sorbents for removing pharmaceutical contaminants from aqueous environments. The main focus was on sorption mechanisms, including electrostatic interactions (caused by protonation of amino groups in an acidic environment), hydrogen bonds, π-π interactions, metal ion chelation, and hydrophobic effects. The influence of key factors such as pH, sorbent characteristics, temperature, and contaminant concentration on the efficiency of the process is also important. It has been noted that modification of chitosan (e.g., cross-linking) and combination with other materials, especially carbon-containing ones, allows for targeted improvement in its adsorption capacity and selectivity.

## 6. Problems and Future Prospects

The release of pharmaceuticals and their metabolites into the environment, including water, is increasing for a variety of reasons due to inadequately designed wastewater treatment processes. This problem is recognised as an environmental issue and is associated with risks to human health. The researchers are proposing and investigating the most efficient and cost-effective ways to remove pharmaceuticals from wastewater. However, research articles often do not reflect how pharmaceutical drugs are actually consumed by humans, utilised, and accumulated in the environment.

In the preparation of adsorbents using chitosan, the authors have usually investigated the factors that influence the adsorption capacity. These are pH, temperature, adsorbent dosage, contact time, initial contaminant concentration, adsorbent form, and ionic strength. Almost all the papers investigated the kinetics and thermodynamics of the process and determined adsorption isotherms. From the obtained results, it was not always possible to draw a definite conclusion regarding the adsorption mechanism.

The determination of the adsorption mechanism of polysaccharide-based adsorbents, including chitosan, is a difficult task. The complexity consists of the features of polysaccharides, namely the presence of reactive functional groups, their distribution, heterogeneity of chemical structure, etc.

From the data obtained by researchers under laboratory conditions, it is clear that only some adsorbents are effective when operating in a natural environment at pH 7.0, constant temperature, and short contact time. Adsorbents lose their effectiveness after several adsorption-regeneration cycles, usually not more than five. Their recycling poses environmental problems. The solution can be found in the use of biodegradable materials, such as polysaccharides and composites based on them [94].

Moreover, the transformation of each specific process from laboratory to pilot process is a complex task and requires additional research [3]. Full-scale studies require the use of computer modelling techniques, surface response methodology, etc. [95,96]. In addition, there is limited data on the adsorption of pollutants from mixtures of real industrial effluents [2,97].

The combination of carbon materials (biochar, activated carbon, and, in particular, graphene nanostructures) with chitosan is a promising direction for the creation of a new generation of adsorbents for the purification of wastewater from pharmaceutical pollutants. This is due to the high adsorption characteristics of such composite materials, which are determined by their improved structural and physicochemical properties, and significant potential for regeneration. However, when developing such materials, it is necessary to carefully monitor their safety and toxicity.

## 7. Conclusions

Pharmaceuticals and their metabolites, which are found in wastewater around the world, pose a growing environmental threat. Their persistence, biological activity even at low concentrations, and potential negative impact on human health and aquatic ecosystems require the development and application of effective treatment methods.

Existing wastewater treatment systems often fail to completely remove a wide range of pharmaceutical micropollutants, especially antibiotics. This leads to their accumulation in the environment, as confirmed by numerous studies in different countries. Among the various treatment technologies, adsorption stands out as one of the most effective, versatile, and cost-effective methods, especially when using inexpensive and renewable adsorbents.

Chitosan, a natural biopolymer, is a highly promising basis for the creation of adsorbents due to its biodegradability, non-toxicity, the presence of reactive functional groups (-NH_2_, -OH), and availability. However, its direct application is limited by low mechanical strength, low specific surface area, and sensitivity to pH.

To overcome the disadvantages of pure chitosan, its modified forms are being actively developed and researched, such as copolymers, chitosan derivatives, and composites, including those with inorganic and carbon materials. Due to a number of advantages, adsorbents based on modified chitosan, especially in the form of composites with carbon and magnetic materials, appear to be an extremely promising and environmentally friendly direction for the creation of effective systems for the purification of wastewater from pharmaceutical micropollutants.

## Figures and Tables

**Table 1:** Some of the pharmaceuticals and their concentrations in various types of water. Adapted from [7].

Water Types	Country, Location	Source	Sulfamethoxazole	Ibuprofen	Carbamazepine
			Concentration, ng/L		
Wastewater Sources	USA (Skaneateles Lake, New York)	Septic effluent	0–37.700	0–10.600	0–2.04
	Portugal (Coimbra)	WWTP influents	529–1662	0–4926	437–673
	China (Shanghai)	Wastewater influents	-	-	45.2
Surface Water Sources	USA (Skaneateles Lake, New York)	Lake water	0–3.21	0–4.98	0–0.17
	Portugal (Lis river)	River	43	1317	-
	China (Chongqing)	River	0.44–115.3	0.86–115.8	0.41–10.3
Treated Waters	USA (Skaneateles Lake, New York)	Tap water	ND–0.39	ND−1.16	1.05
	Portugal	-	-	-	-
	China (Beijing)	Tap water	<LOD *–1.81	<LOD–17.17 **	0.51–38.24

* Limit of detection, ** Drinking water.

**Table 2:** Chitosan and chitosan composites for the removal of some antibiotics by adsorption method.

Adsorbents	Antibiotic	Adsorption Capacity, mg/g	Reference
Chitosan, particles	Rifampicin	66.91	[56]
	Streptomycin	11.0	
Chitosan/PAA	Enrofloxacin	387.7	[58]
Metal–organic framework (MIL-53)/Chitosan	Doxycycline	264.0	[59]
Chitosan/magnetic Fe_3_O_4_ nanoparticles	Metronidazole	97.0	[60]
AgZnFe_2_O_4_/chitosan	Metronidazole	7.28	[61]
Chitosan/biochar, particles	Enrofloxacin	100.43	[62]
Magnetic activated charcoal/chitosan	Amoxycycline	526.31	[63]
Gelatin/chitosan/graphene oxide	Ofloxacin	8.3	[64]
Carboxymethylcellulose/carboxyalkylchitosan cross-linked with genipin/sulphated graphene oxide	Sulfapyridine	161.89	[65]
	Sulfamethoxazole	312.28	
Chitosan/Fe_3_O_4_/reduced graphene oxide	Cefixime	29.99	[66]
Chitosan-olive leaf biomass composites	Amoxicillin	0.04	[67]
Graphene oxide/chitosan	Levofloxacin	51.5	[68]
NiFe_2_O_4_-COF-chitosan-terephthalaldehyde nanocomposites film	Cefotaxime	309.26	[69]

**Table 3:** Composites with chitosan for removal of tetracycline (TC) and ciprofloxacin (CIP) by adsorption method.

Adsorbents	Adsorption Capacity, mg/g		Reference
	TC	CIP	
CTS-PAA, chitosan, polyacrylic acid	-	267.7	[58]
SFMOF/BM, chitosan, organometallic framework, 3-aminopropyltrimethoxysilane	388	-	[59]
Cu(II)-chelated cryogel of N-(2-carboxyethyl) chitosan	-	280	[76]
CTM/Fe_3_O_4_, chitosan, thiobarbituric acid, malonic dialdehyde, Fe_3_O_4_ nanoparticles	215.31	-	[77]
CS/Fe_3_O_4_ (M), chitosan, iron oxide	211.21	-	[78]
CuCoFe_2_O_4_/Ch, chitosan, copper, cobalt, iron 3^+^ chlorides	11.63	-	[79]
NiFe_2_O_4_-COF-chitosan-terephthalaldehyde nanocomposites film	388.52	-	[69]
Chitosan/BC (M), chitosan/biochar	183.01	-	[80]
Chitosan/BC, chitosan/biochar	-	>76	[81]
Chitosan/BC, chitosan/biochar	-	106.038	[62]
Chitosan/AC, (M *) chitosan/activated charcoal	-	90.10	[63]
Chitosan/GO (M), chitosan/graphene oxide	495.64		[65]
Chitosan/GO (M), chitosan/graphene oxide	-	282.9	[82]
Chitin/GO, chitin/graphene oxide	-	73.0	[83]

* magnetic.

## Data Availability

No new data were created or analysed in this study.

## References

[1] Wilkinson J.L., Boxall A.B.A., Kolpin D.W., Leung K.M.Y., Lai R.W.S., Wong D., Ntchantcho R., Pizarro J., Mart J., Echeverr S. (2022). Pharmaceutical pollution of the world’ s rivers. Proc. Natl. Acad. Sci. USA.

[2] González Peña O.I., López Zavala M.Á., Cabral Ruelas H. (2021). Pharmaceuticals market, consumption trends and disease incidence are not driving the pharmaceutical research on water and wastewater. Int. J. Environ. Res. Public Health.

[3] Quesada H.B., Baptista A.T.A., Cusioli L.F., Seibert D., de Oliveira Bezerra C., Bergamasco R. (2019). Surface water pollution by pharmaceuticals and an alternative of removal by low-cost adsorbents: A review. Chemosphere.

[4] Kumar V., Lakkaboyana S.K., Sharma N., Chakraborty P., Umesh M., Pasrija R., Thomas J., Kalebar V.U., Jayaraj I., Awasthi M.K. (2023). A critical assessment of technical advances in pharmaceutical removal from wastewater—A critical review. Case Stud. Chem. Environ. Eng..

[5] Bottoni P., Caroli S. (2018). Presence of residues and metabolites of pharmaceuticals in environmental compartments, food commodities and workplaces: A review spanning the three-year period 2014–2016. Microchem. J..

[6] Kumar V.S., Dhivakar M., Nagamani S., Dhanalakshmi A., Leema M.A. (2024). Removal of pharmaceuticals from wastewater: A review of different adsorptive approaches. Glob. Nest J..

[7] Patel M., Kumar R., Kishor K., Mlsna T., Pittman C.U., Mohan D. (2019). Pharmaceuticals of emerging concern in aquatic systems: Chemistry, occurrence, effects, and removal methods. Chem. Rev..

[8] Paíga P., Correia-Sá L., Correia M., Figueiredo S., Vieira J., Jorge S., Silva J.G., Delerue-Matos C. (2024). Temporal Analysis of Pharmaceuticals as Emerging Contaminants in Surface Water and Wastewater Samples: A Case Study. J. Xenobiotics.

[9] Otero M., Coimbra R.N. (2025). Polymeric Materials for Wastewater Treatment Applications. Polymers.

[10] Wada O.Z., Olawade D.B. (2025). Recent occurrence of pharmaceuticals in freshwater, emerging treatment technologies, and future considerations: A review. Chemosphere.

[11] Afonso-Olivares C., Sosa-Ferrera Z., Santana-Rodríguez J.J. (2017). Occurrence and environmental impact of pharmaceutical residues from conventional and natural wastewater treatment plants in Gran Canaria (Spain). Sci. Total Environ..

[12] Afonso-Olivares C., Čadková T., Sosa-Ferrera Z., Santana-Rodríguez J.J., Nováková L. (2017). Simplified solid-phase extraction procedure combined with liquid chromatography tandem–mass spectrometry for multiresidue assessment of pharmaceutical compounds in environmental liquid samples. J. Chromatogr. A.

[13] Escher B.I., Baumgartner R., Koller M., Treyer K., Lienert J., McArdell C.S. (2011). Environmental toxicology and risk assessment of pharmaceuticals from hospital wastewater. Water Res..

[14] Praveena S.M., Shaifuddin S.N.M., Sukiman S., Nasir F.A.M., Hanafi Z., Kamarudin N., Ismail T.H.T., Aris A.Z. (2018). Pharmaceuticals residues in selected tropical surface water bodies from Selangor (Malaysia): Occurrence and potential risk assessments. Sci. Total Environ..

[15] Al-Qaim F.F., Abdullah M.P., Othman M.R., Latip J., Zakaria Z. (2014). Multi-residue analytical methodology-based liquid chromatography-time-of-flight-mass spectrometry for the analysis of pharmaceutical residues in surface water and effluents from sewage treatment plants and hospitals. J. Chromatogr. A.

[16] Asghar M.A., Zhu Q., Sun S., Peng Y., Shuai Q. (2018). Suspect screening and target quantification of human pharmaceutical residues in the surface water of Wuhan, China, using UHPLC-Q-Orbitrap HRMS. Sci. Total Environ..

[17] Commission Implementing Decision (EU) 2022/1307 of 22 July 2022 Establishing a Watch List of Substances for Union-Wide Monitoring in the Field of Water Policy Pursuant to Directive 2008/105/EC of the European Parliament and of the Council (Notified Under Document C(2022) 5098). https://euroalert.net/en/oj/105661/commission-implementing-decision-eu-2022-1307-of-22-july-2022-establishing-a-watch-list-of-substances-for-union-wide-monitoring-in-the-field-of-water-policy-pursuant-to-directive-2008-105-ec-of-the-european-parliament-an.

[18] Kokoszka K., Wilk J., Felis E., Bajkacz S. (2021). Application of UHPLC-MS/MS method to study occurrence and fate of sulfonamide antibiotics and their transformation products in surface water in highly urbanized areas. Chemosphere.

[19] Guo H., Li D., Li Z., Lin S., Wang Y., Pan S., Han J. (2021). Promoted elimination of antibiotic sulfamethoxazole in water using sodium percarbonate activated by ozone: Mechanism, degradation pathway and toxicity assessment. Sep. Purif. Technol..

[20] Eniola J.O., Kumar R., Barakat M.A., Rashid J. (2022). A review on conventional and advanced hybrid technologies for pharmaceutical wastewater treatment. J. Clean. Prod..

[21] Rashid R., Shafiq I., Akhter P., Iqbal M.J., Hussain M. (2021). A state-of-the-art review on wastewater treatment techniques: The effectiveness of adsorption method. Environ. Sci. Pollut. Res..

[22] Wang J., Liu X. (2021). Forward osmosis technology for water treatment: Recent advances and future perspectives. J. Clean. Prod..

[23] Tiwari B., Sellamuthu B., Ouarda Y., Drogui P., Tyagi R.D., Buelna G. (2017). Review on fate and mechanism of removal of pharmaceutical pollutants from wastewater using biological approach. Bioresour. Technol..

[24] Alfonso-Muniozguren P., Serna-Galvis E.A., Bussemaker M., Torres-Palma R.A., Lee J. (2021). A review on pharmaceuticals removal from waters by single and combined biological, membrane filtration and ultrasound systems. Ultrason. Sonochem..

[25] Miklos D.B., Remy C., Jekel M., Linden K.G., Drewes J.E., Hübner U. (2018). Evaluation of advanced oxidation processes for water and wastewater treatment—A critical review. Water Res..

[26] Qin Z., Liu S., Liang S.X., Kang Q., Wang J., Zhao C. (2016). Advanced treatment of pharmaceutical wastewater with combined micro-electrolysis, Fenton oxidation, and coagulation sedimentation method. Desalin. Water Treat..

[27] Radjenovic J., Sedlak D.L. (2015). Challenges and Opportunities for Electrochemical Processes as Next-Generation Technologies for the Treatment of Contaminated Water. Environ. Sci. Technol..

[28] Prada-Vásquez M.A., Estrada-Flórez S.E., Serna-Galvis E.A., Torres-Palma R.A. (2021). Developments in the intensification of photo-Fenton and ozonation-based processes for the removal of contaminants of emerging concern in Ibero-American countries. Sci. Total Environ..

[29] Azmi L.S., Jabit N.A., Ismail S., Ku Ishak K.E.H., Abdullah T.K. (2025). Membrane filtration technologies for sustainable industrial wastewater treatment: A review of heavy metal removal. Desalin. Water Treat..

[30] Kafle S.R., Adhikari S., Shrestha R., Ban S., Khatiwada G., Gaire P., Tuladhar N., Jiang G., Tiwari A. (2024). Advancement of membrane separation technology for organic pollutant removal. Water Sci. Technol..

[31] Fonseca Couto C., Lange L.C., Santos Amaral M.C. (2018). A critical review on membrane separation processes applied to remove pharmaceutically active compounds from water and wastewater. J. Water Process Eng..

[32] Boleda M.R., Galceran M.T., Ventura F. (2011). Behavior of pharmaceuticals and drugs of abuse in a drinking water treatment plant (DWTP) using combined conventional and ultrafiltration and reverse osmosis (UF/RO) treatments. Environ. Pollut..

[33] Silva L.L.S., Moreira C.G., Curzio B.A., da Fonseca F.V. (2017). Micropollutant Removal from Water by Membrane and Advanced Oxidation Processes—A Review. J. Water Resour. Prot..

[34] Martins T.A.E., Muñoz Sierra J.D., Nieuwlands J.A., Lousada-Ferreira M., Amaral L. (2024). Micropollutant biotransformation under different redox conditions in PhoRedox conventional activated sludge systems. Environ. Technol. Innov..

[35] Martin M., Wu J., Rich S.L., Richardson R.E., Helbling D.E. (2024). Differential biotransformation of micropollutants in conventional activated sludge and up-flow anaerobic sludge blanket processes. Environ. Sci. Water Res. Technol..

[36] Zheng W., Wen X., Zhang B., Qiu Y. (2019). Selective effect and elimination of antibiotics in membrane bioreactor of urban wastewater treatment plant. Sci. Total Environ..

[37] Pervez M.N., Balakrishnan M., Hasan S.W., Choo K.H., Zhao Y., Cai Y., Zarra T., Belgiorno V., Naddeo V. (2020). A critical review on nanomaterials membrane bioreactor (NMS-MBR) for wastewater treatment. NPJ Clean Water.

[38] Satyam S., Patra S. (2024). Innovations and challenges in adsorption-based wastewater remediation: A comprehensive review. Heliyon.

[39] Dutta S., Gupta B., Srivastava S.K., Gupta A.K. (2021). Recent advances on the removal of dyes from wastewater using various adsorbents: A critical review. Mater. Adv..

[40] Natarajan R., Saikia K., Ponnusamy S.K., Rathankumar A.K., Rajendran D.S., Venkataraman S., Tannani D.B., Arvind V., Somanna T., Banerjee K. (2022). Understanding the factors affecting adsorption of pharmaceuticals on different adsorbents—A critical literature update. Chemosphere.

[41] Hosseinian Naeini A., Hosseini Moradi S.A. (2023). Adsorption Method for Removal of Pharmaceuticals from Wastewater: Review. Iran. J. Mater. Sci. Eng..

[42] Atheena P.V., Basawa R., Raval R. (2024). Advancing wastewater treatment: Chitin and derivatives for PPCP contaminant mitigation. Polym. Bull..

[43] Magesh N., Annam Renita A., Senthil Kumar P. (2020). Practice on treating pharmaceutical compounds (antibiotics) present in wastewater using biosorption techniques with different biowaste compounds. A review. Environ. Prog. Sustain. Energy.

[44] Fekete E., Csiszár E. (2024). Chitosan–Alginate Gels for Sorption of Hazardous Materials: The Effect of Chemical Composition and Physical State. Int. J. Mol. Sci..

[45] Bhatt P., Joshi S., Urper Bayram G.M., Khati P., Simsek H. (2023). Developments and application of chitosan-based adsorbents for wastewater treatments. Environ. Res..

[46] Benettayeb A., Ghosh S., Usman M., Seihoub F.Z., Sohoo I., Chia C.H., Sillanpää M. (2022). Some Well-Known Alginate and Chitosan Modifications Used in Adsorption: A Review. Water.

[47] Wong S., Ghafar N.A., Ngadi N., Razmi F.A., Inuwa I.M., Mat R., Amin N.A.S. (2020). Effective removal of anionic textile dyes using adsorbent synthesized from coffee waste. Sci. Rep..

[48] Turk Sekulic M., Boskovic N., Slavkovic A., Garunovic J., Kolakovic S., Pap S. (2019). Surface functionalised adsorbent for emerging pharmaceutical removal: Adsorption performance and mechanisms. Process Saf. Environ. Prot..

[49] Sarode S., Upadhyay P., Khosa M.A., Mak T., Shakir A., Song S., Ullah A. (2019). Overview of wastewater treatment methods with special focus on biopolymer chitin-chitosan. Int. J. Biol. Macromol..

[50] Varlamov V.P., Il’ina A.V., Shagdarova B.T., Lunkov A.P., Mysyakina I.S. (2020). Chitin/Chitosan and Its Derivatives: Fundamental Problems and Practical Approaches. Biochemistry.

[51] Chen X., Yang H., Zhong Z., Yan N. (2017). Base-catalysed, one-step mechanochemical conversion of chitin and shrimp shells into low molecular weight chitosan. Green Chem..

[52] Pandey R., Mathur G. (2024). Current Trends in Chitosan Functionalization Methods and Their Applications. Starch/Staerke.

[53] Kaczorowska M.A., Bożejewicz D. (2024). The Application of Chitosan-Based Adsorbents for the Removal of Hazardous Pollutants from Aqueous Solutions—A Review. Sustainability.

[54] da Silva Alves D.C., Healy B., Pinto L.A.d.A., Cadaval T.R.S., Breslin C.B. (2021). Recent developments in Chitosan-based adsorbents for the removal of pollutants from aqueous environments. Molecules.

[55] Dago-Serry Y., Maroulas K.N., Tolkou A.K., Kokkinos N.C., Kyzas G.Z. (2024). How the chitosan structure can affect the adsorption of pharmaceuticals from wastewaters: An overview. Carbohydr. Polym. Technol. Appl..

[56] Shahrin E.W.E.S., Narudin N.A.H., Shahri N.N.M., Nur M., Lim J.W., Bilad M.R., Mahadi A.H., Hobley J., Usman A. (2023). A comparative study of adsorption behavior of rifampicin, streptomycin, and ibuprofen contaminants from aqueous solutions onto chitosan: Dynamic interactions, kinetics, diffusions, and mechanisms. Emerg. Contam..

[57] Rizzi V., Gubitosa J., Fini P., Romita R., Nuzzo S., Gabaldón J.A., Gorbe M.I.F., Gómez-Morte T., Cosma P. (2020). Chitosan film as recyclable adsorbent membrane to remove/recover hazardous pharmaceutical pollutants from water: The case of the emerging pollutant Furosemide. J. Environ. Sci. Health—Part A Toxic/Hazardous Subst. Environ. Eng..

[58] Wang N., Xiao W., Niu B., Duan W., Zhou L., Zheng Y. (2019). Highly efficient adsorption of fluoroquinolone antibiotics using chitosan derived granular hydrogel with 3D structure. J. Mol. Liq..

[59] Allahbakhshi M., Mahmoodi N.M., Mosaferi M., Kazemian H., Aslani H. (2022). Synthesis of functionalized metal-organic framework metal-organic framework (MIL-53)/Chitosan for removing dye and pharmaceuticals. Surf. Interfaces.

[60] Asgari E., Sheikhmohammadi A., Yeganeh J. (2020). Application of the Fe3O4-chitosan nano-adsorbent for the adsorption of metronidazole from wastewater: Optimization, kinetic, thermodynamic and equilibrium studies. Int. J. Biol. Macromol..

[61] Rajabi S., Derakhshan Z., Hashemi M., Feilizadeh M., Heidari Kochaki S., Hashemi H., Salehi M., Zare A., Shourabi N.S., Moradalizadeh S. (2024). Metronidazole adsorption by bio-synthesized silver-zinc ferrite nanoadsorbent in presence of chitosan from aqueous media: Response surface methodology. Appl. Water Sci..

[62] Nguyen H.T., Phuong V.N., Van T.N., Thi P.N., Dinh Thi Lan P., Pham H.T., Cao H.T. (2020). Low-cost hydrogel derived from agro-waste for veterinary antibiotic removal: Optimization, kinetics, and toxicity evaluation. Environ. Technol. Innov..

[63] Danalıoğlu S.T., Bayazit Ş.S., Kerkez Kuyumcu Ö., Salam M.A. (2017). Efficient removal of antibiotics by a novel magnetic adsorbent: Magnetic activated carbon/chitosan (MACC) nanocomposite. J. Mol. Liq..

[64] Kovtun A., Campodoni E., Favaretto L., Zambianchi M., Salatino A., Amalfitano S., Navacchia M.L., Casentini B., Palermo V., Sandri M. (2020). Multifunctional graphene oxide/biopolymer composite aerogels for microcontaminants removal from drinking water. Chemosphere.

[65] Liu Y., Liu R., Li M., Yu F., He C. (2019). Removal of pharmaceuticals by novel magnetic genipin-crosslinked chitosan/graphene oxide-SO3H composite. Carbohydr. Polym..

[66] Ciğeroğlu Z., Küçükyıldız G., Erim B., Alp E. (2021). Easy preparation of magnetic nanoparticles-rGO-chitosan composite beads: Optimization study on cefixime removal based on RSM and ANN by using Genetic Algorithm Approach. J. Mol. Struct..

[67] Alakayleh Z. (2025). From inactive biomass in removing amoxicillin to new active chitosan-biomass composite adsorbents. Results Eng..

[68] Miao P., Gao J., Han X., Zhao Y., Chen T. (2024). Adsorption of Levofloxacin onto Graphene Oxide/Chitosan Composite Aerogel Microspheres. Gels.

[69] Li Z., Liu Y., Zou S., Lu C., Bai H., Mu H., Duan J. (2020). Removal and adsorption mechanism of tetracycline and cefotaxime contaminants in water by NiFe2O4-COF-chitosan-terephthalaldehyde nanocomposites film. Chem. Eng. J..

[70] Daikh S., Ouis D., Benyoucef A., Mouffok B. (2022). Equilibrium, kinetic and thermodynamic studies for evaluation of adsorption capacity of a new potential hybrid adsorbent based on polyaniline and chitosan for Acetaminophen. Chem. Phys. Lett..

[71] Alamir H.T.A., Alalaq I.S., Naser S.T., Abdulamer R.S., Abid M.M., Dawood I.I., Idan A.H. (2024). Fabrication of Polymeric Chitosan-g-P (ITA-co-AM) Nanocomposite Using Copolymerization and Application to Removal Metformin Drug from Aqueous Solution. Asian J. Green Chem..

[72] Ma J., Lei Y., Khan M.A., Wang F., Chu Y., Lei W., Xia M., Zhu S. (2019). Adsorption properties, kinetics & thermodynamics of tetracycline on carboxymethyl-chitosan reformed montmorillonite. Int. J. Biol. Macromol..

[73] Sousa J.F.M., Murtinho D., Valente A.J.M., Marques J.M.C. (2025). On the Mechanism of Interactions Between Tetracycline and New Chitosan-Based Materials: Experimental Development Guided by Computational Methods. J. Polym. Sci..

[74] Khierallah A.H.I., Bates I.I.C., Chabot B., Lajeunesse A. (2021). Adsorption of Pharmaceutical Contaminants from Aqueous Solutions Using N,O-Carboxymethyl Chitosan/Polyethylene Oxide (PEO) Electrospun Nanofibers. J. Mater. Sci. Chem. Eng..

[75] Khierallah A.H.I., Bouazza A.H., Montplaisir D. (2023). Nanofibrous material of n-succinyl chitosan/polyethylene oxide in the removal of emerging pharmaceuticals from aqueous solution by adsorption/desorption method. BioResources.

[76] Privar Y., Shashura D., Pestov A., Modin E., Baklykov A., Marinin D., Bratskaya S. (2019). Metal-chelate sorbents based on carboxyalkylchitosans: Ciprofloxacin uptake by Cu(II) and Al(III)-chelated cryogels of N-(2-carboxyethyl)chitosan. Int. J. Biol. Macromol..

[77] Ahamad T., Naushad M., Al-Shahrani T., Al-hokbany N., Alshehri S.M. (2020). Preparation of chitosan based magnetic nanocomposite for tetracycline adsorption: Kinetic and thermodynamic studies. Int. J. Biol. Macromol..

[78] da Silva Bruckmann F., Schnorr C.E., da Rosa Salles T., Nunes F.B., Baumann L., Müller E.I., Silva L.F.O., Dotto G.L., Bohn Rhoden C.R. (2022). Highly Efficient Adsorption of Tetracycline Using Chitosan-Based Magnetic Adsorbent. Polymers.

[79] Nasiri A., Rajabi S., Amiri A., Fattahizade M., Hasani O., Lalehzari A., Hashemi M. (2022). Adsorption of tetracycline using CuCoFe_2_O_4_@Chitosan as a new and green magnetic nanohybrid adsorbent from aqueous solutions: Isotherm, kinetic and thermodynamic study. Arab. J. Chem..

[80] Liu J., Zhou B., Zhang H., Ma J., Mu B., Zhang W. (2019). A novel Biochar modified by Chitosan-Fe/S for tetracycline adsorption and studies on site energy distribution. Bioresour. Technol..

[81] Afzal M.Z., Sun X.F., Liu J., Song C., Wang S.G., Javed A. (2018). Enhancement of ciprofloxacin sorption on chitosan/biochar hydrogel beads. Sci. Total Environ..

[82] Wang F., Yang B., Wang H., Song Q., Tan F., Cao Y. (2016). Removal of ciprofloxacin from aqueous solution by a magnetic chitosan grafted graphene oxide composite. J. Mol. Liq..

[83] González J.A., Bafico J.G., Villanueva M.E., Giorgieri S.A., Copello G.J. (2018). Continuous flow adsorption of ciprofloxacin by using a nanostructured chitin/graphene oxide hybrid material. Carbohydr. Polym..

[84] Machado T.S., Crestani L., Marchezi G., Melara F., de Mello J.R., Dotto G.L., Piccin J.S. (2022). Synthesis of glutaraldehyde-modified silica/chitosan composites for the removal of water-soluble diclofenac sodium. Carbohydr. Polym..

[85] Gencer Balkis B., Aksu A., Ersoy Korkmaz N., Taskin O.S., Celen C., Caglar Balkis N. (2024). Synthesis of silica-chitosan nanocomposite for the removal of pharmaceuticals from the aqueous solution. Int. J. Environ. Sci. Technol..

[86] Lai L.W., Teh L.P., Timmiati S.N., Kamarudin N.H.N., Setiabudi H.D. (2023). A sustainable solution for diclofenac adsorption: Chitosan-modified fibrous silica KCC-1 adsorbent. J. Environ. Chem. Eng..

[87] Petit C. (2018). Present and future of MOF research in the field of adsorption and molecular separation. Curr. Opin. Chem. Eng..

[88] Rao R.S., Kumar C.G., Prakasham R.S., Hobbs P.J. (2008). The Taguchi methodology as a statistical tool for biotechnological applications: A critical appraisal. Biotechnol. J..

[89] Ahmed M.J., Hameed B.H., Hummadi E.H. (2020). Review on recent progress in chitosan/chitin-carbonaceous material composites for the adsorption of water pollutants. Carbohydr. Polym..

[90] Delhiraja K., Vellingiri K., Boukhvalov D.W., Philip L. (2019). Development of Highly Water Stable Graphene Oxide-Based Composites for the Removal of Pharmaceuticals and Personal Care Products. Ind. Eng. Chem. Res..

[91] Liu Y., Nie P., Yu F. (2021). Enhanced adsorption of sulfonamides by a novel carboxymethyl cellulose and chitosan-based composite with sulfonated graphene oxide. Bioresour. Technol..

[92] Shen M., Yu B., Hu Y., Liu Z., Zhao K., Li C., Li M., Lyu C., Lu H., Zhong S. (2023). Occurrence and Health Risk Assessment of Sulfonamide Antibiotics in Different Freshwater Fish in Northeast China. Toxics.

[93] Kulyk B., Freitas M.A., Santos N.F., Mohseni F., Carvalho A.F., Yasakau K., Fernandes A.J.S., Bernardes A., Figueiredo B., Silva R. (2022). A critical review on the production and application of graphene and graphene-based materials in anti-corrosion coatings. Crit. Rev. Solid State Mater. Sci..

[94] Qi X., Tong X., Pan W., Zeng Q., You S., Shen J. (2021). Recent advances in polysaccharide-based adsorbents for wastewater treatment. J. Clean. Prod..

[95] dos Santos J.M.N., Lima É., Dotto G.L. (2022). Basic fundamentals of adsorption modeling for removal of pesticides from water and wastewater. Pesticides Remediation Technologies from Water and Wastewater.

[96] Teixeira C.C., Pereira A.K.d.S., Cavallini G.S., Pereira D.H. (2025). Triclosan Adsorption on Chitosan: Computational Study of Molecular Interactions and Potential for Environmental Remediation. Polymers.

[97] Elwakeel K.Z., Mohammad R.M., Alghamdi H.M., Elgarahy A.M. (2025). Hybrid adsorbents for pollutants removal: A comprehensive review of chitosan, glycidyl methacrylate and their composites. J. Mol. Liq..

